# The International Guideline for the Definition, Classification, Diagnosis and Management of Urticaria

**DOI:** 10.1111/all.70210

**Published:** 2026-02-06

**Authors:** Torsten Zuberbier, Zainab AbdulHameed Ansari, Amir H. Abdul Latiff, M. M. Abuzakouk, Maria Socorro Agcaoili‐De Jesus, Rosana C. Agondi, Mona Al‐Ahmad, Abdullah A. Alangari, H. Alhameli, Cesar D. Alonso Bello, Saad Alshareef, Salem Al‐Tamemi, Sabine Altrichter, Humaid Al Wahshi, S. Aquilina, M. Araújo, Rand Arnaout, Riccardo Asero, Barbara Ballmer‐Weber, Christine Bangert, Andrea Bauer, Moshe Ben‐Shoshan, Jonathan A. Bernstein, Carsten Bindslev‐Jensen, Mojca Bizjak, Isabelle Boccon‐Gibod, Hanna Bonnekoh, Laurence Bouillet, Knut Brockow, Zenon Brzoza, M. Bulatović Ćalasan, Adeeb Bulkhi, Thomas Buttgereit, Anette Bygum, Teresa Caballero, Oscar Calderon, R. Campos, Mauro Cancian, Emily Carne, Mary Anne Castor, Inmaculada Cerecedo, T. Çetinarslan, Ivan Cherrez‐Ojeda, N. Chkhikvadze, Herberto J. Chong‐Neto, Karen Choo, George Christoff, Chia‐Yu Chu, K. Ciupka, Niall Conlon, Célia Costa, Timothy J Craig, Paulo Criado, Inna Danilycheva, Razvigor Darlenski, Erika De Arruda Chaves, Laurence de Montjoye, M.‐S. Doutre, Aurelie Du‐Thanh, Didier Ebo, S. Elkhalifa, S. Elmariah, Tariq El‐Shanawany, Luis F. Ensina, Ragip Ertaş, Roberta Fachini Jardim Criado, Marta Ferrer, Silvia Ferrucci, Jie Shen Fok, Daria Fomina, Luz Fonacier, Ghada Fouda, I. Francescantonio, Atsushi Fukunaga, César A. Galvan Calle, Elizabeth Garcia, Krisztian Gáspár, Aslı Gelincik, Songmei Geng, Kiran Godse, Margarida Gonçalo, Maia Gotua, C. Grattan, Martine Grosber, Guillermo Guidos Fogelbach, Mar Guilarte, R. Guillod, Eckard Hamelmann, Jason Hawkes, Koremasa Hayama, Ruben Heuer, Michihiro Hide, Wolfram Hoetzenecker, Naoko Inomata, Hye‐Ryun Kang, Allen P. Kaplan, Alexander Kapp, M. Karam, Alicja Kasperska‐Zajac, Constance H. Katelaris, Aharon Kessel, Maryam Khoshkhui, Brian Kim, Tamar Kinaciyan, Emek Kocatürk, Marta Kolacinska‐Flont, Pavel Kolkhir, George N. Konstantinou, Mitja Kosnik, Dorota Krasowska, Kanokvalai Kulthanan, Muthu S. Kumaran, Izabela Kuprys‐Lipinska, Moisés Labrador, Jose Ignacio Larco, Désirée Larenas‐Linnemann, Elena Latysheva, E. Lazaridou, Philip H. Li, H. Lima, Undine Lippert, Markus Magerl, Michael Makris, João Alves Marcelino, Angelo V. Marzano, Iris Medina, Raisa Meshkova, D. Micallef, Ramzy Mohammed Ali, Charlotte G. Mortz, Melba Munoz, Hanneke N. G. Oude Elberink, Alla Nakonechna, Iman Nasr, Alexander Nast, Elena Netchiporouk, Eustachio Nettis, Sandra Nieto, Isabel Ogueta Canales, T.‐L. Okas, Raquel L. Orfali, Esen Özkaya, Claudio Parisi, A. Pennitz, Ruby Pawankar, Manuel P. Pereira, Jonny Peter, Elena Petkova, P. D. Pigatto, Indrashis Podder, T. Popov, Grzegorz Porebski, Polina Pyatilova, German D. Ramon, Hector A. Ratti Sisa, Marysia Stella Recto, Krista Ress, Katie Ridge, Marc Riedl, Carla Ritchie, Nelson Rosario Filho, Isabel Rosmaninho, Michael Rudenko, Maia Rukhadze, Krzysztof Rutkowski, Vito Sabato, Umit M. Sahiner, Sarbjit Saini, F. Saleh Al Sabbagh, Andac Salman, Fulvio Salvo, Jorge Sanchez, A. Santucci, Sibylle Schliemann, Peter Schmid‐Grendelmeier, Bulent E. Sekerel, Faradiba Serpa, Farrukh Sheikh, J. Sheikh, H. Shendi, Frank Siebenhaar, M. Sonomjamts, Angele Soria, Bernardo Sousa Pinto, Maria Staevska, Petra Staubach, M. Stephan, Katarina Stevanovic, Luca Stingeni, Marcin Stobiecki, Özlem Su Küçük, Gordon Sussman, Andrea Szegedi, Shunsuke Takahagi, Akio Tanaka, Natasa Teovska Mitrevska, Simon Francis Thomsen, Elias Toubi, Fragkiski Tsatsou, Murat Turk, Zahava Vadasz, Anna Valerieva, Solange Valle, Martijn van Doorn, Beatriz Veleiro Perez, Carolina E. Vera Ayala, Christian Vestergaard, Rafael J. Vieira, C. W. Maruta, Bettina Wedi, Ricardo N. Werner, Evelyn Wen Yee Yap, Paraskevi Xepapadaki, Yi‐Kui Xiang, Young‐Min Ye, Patrick Yong, Gil Yosipovitch, Anna Zalewska‐Janowska, Christoph Zeyen, Zuotao Zhao, Martin Metz, Ana M. Giménez‐Arnau

**Affiliations:** ^1^ Institute of Allergology, Charité—Universitätsmedizin Berlin, Corporate Member of Freie Universität Berlin and Humboldt‐Universität zu Berlin Berlin Germany; ^2^ Fraunhofer Institute for Translational Medicine and Pharmacology ITMP, Immunology and Allergology Berlin Germany; ^3^ Clinical Immunology & Allergy, Internal Medicine, Royal Hospital Oman; ^4^ Pantai Hospital Kuala Lumpur, Allergy & Immunology Centre Malaysia; ^5^ Cleveland Clinic Abu Dhabi, Allergy and Immunology UAE; ^6^ Division of Allergy and Immunology, Department of Medicine University of the Philippines‐Philippine General Hospital Philippines; ^7^ Hospital das Clínicas da Universidade de São Paulo, Clinical Immunology and Allergy Division – Internal Medicine Department Brazil; ^8^ College of Medicine Kuwait University, Kuwait and Al‐Rashed Allergy Center, Ministry of Health Kuwait; ^9^ Allergy and Immunology Unit, Department of Pediatrics, College of Medicine King Saud University Saudi Arabia; ^10^ Hospital Juárez de México, Allergy and Immunology Mexico; ^11^ Allergy Immunology, King Faisal Specialist Hospital and Research Center Saudi Arabia; ^12^ Sultan Qaboos University Hospital, Child Health Oman; ^13^ Omani Society of Allergy & Clinical Immunology Oman; ^14^ Department of Dermatology and Venerology Kepler University Hospital Austria; ^15^ Department of Rheumatology Royal Hospital Muscat Oman; ^16^ Department of Dermatology Mater Dei Hospital Malta; ^17^ Serviço de Imunoalergologia do Hospital Dona Estefânia, CHULC E.P.E Portugal; ^18^ Clinica San Carlo, Ambulatorio di Allergologia Italy; ^19^ Department of Dermatology University Hospital Zurich and Clinic for Dermatology and Allergology Kantonsspital St. Gallen Switzerland; ^20^ Department of Dermatology Medical University of Vienna Austria; ^21^ Department of Dermatology University Hospital Carl Gustav Carus Germany; ^22^ Montreal Children's Hospital Allergy Clinic, Pediatrics, Division of Allergy Immunology and Dermatology Canada; ^23^ University of Cincinnati Department of Internal Medicine, Division of Rheumatology, Allergy and Immunology, Advanced Allergy Services LLC and Bernstein Clinical Research Center LLC USA; ^24^ Allergy Center – ORCA, Department of Dermatology and Allergy Center Denmark; ^25^ Division of Allergy University Clinic of Respiratory and Allergic Diseases Golnik Slovenia; ^26^ Faculty of Medicine University of Ljubljana Slovenia; ^27^ Faculty of Medicine University of Maribor Slovenia; ^28^ National Reference Center for Angiœdema CREAK, Internal Medicine and Clinical Immunology France; ^29^ Department of Dermatology and Allergy Biederstein, Faculty of Medicine and Health Technical University of Munich Germany; ^30^ Department of Internal Diseases, Allergology, Endocrinology and Gastroenterology University of Opole Poland; ^31^ Rheumatology and Clinical Immunology University Medical Center Utrecht the Netherlands; ^32^ Department of Internal Medicine College of Medicine, Umm Al‐Qura University Makkah Saudi Arabia; ^33^ Clinical Institute University of Southern Denmark Denmark; ^34^ Allergy Department, Hospital Universitario La Paz, IdiPAZ, CIBERER Spain; ^35^ Clinica Sanna el Golf, San Isidro, Allergy Unit Peru; ^36^ Complexo Hospitalar Universitário, Universidade Federal da Bahia, Internal Medicine Brazil; ^37^ Departmental Allergy Unit, Department of Systems Medicine Italy; ^38^ Allergy Service for Wales UK; ^39^ Dermatology and Venereology Manisa Celal Bayar University Turkey; ^40^ Espiritu Santo University Ecuador; ^41^ Respiralab Research Center Ecuador; ^42^ Allergy and Immunology, Center of Allergy and Immunology Tbilisi Georgia; ^43^ Serviço de Alergia e Imunologia, Complexo Hospital de Clínicas, Universidade Federal do Paraná, Pediatrics Brazil; ^44^ Department of Dermatology Urticaria Clinic, Singapore General Hospital Singapore; ^45^ Medical Center Excelsior Ltd. Bulgaria; ^46^ Immunology Clinic, Department of Dermatology National Taiwan University Hospital Taiwan; ^47^ Department of Clinical and Diagnostic Immunology St. James's Hospital Ireland; ^48^ Serviço de Imunoalergologia, Unidade Local de Saúde Santa Maria (ULSSM) Lisboa Portugal; ^49^ Pediatrics, Ob/Gyn and Biomedical Sciences Penn State University Hershey Pennsylvania USA; ^50^ Vinmec International Hospital, Times City Hanoi Vietnam; ^51^ Alergoskin Alergia e Dermatologia SS Ltda, and Centro Universitário Faculdade de Medicina do ABC, Dermatology Brazil; ^52^ NRC Institute of Immunology FMBA of Russia, Allegology Russia; ^53^ Department of Dermatology and Venereology Excelsior Med Center/Acibadem Cityclinic Tokuda Hospital (Inpatients) Bulgaria; ^54^ Allergy and Immunology Clinica Anglo Americana Peru; ^55^ Dermatology Department, Cliniques Universitaires Saint‐Luc Université Catholique de Louvain Belgium; ^56^ Bordeaux University Hospital, Dermatology France; ^57^ Dermatology CHU de Montpellier France; ^58^ Immunology Allergology Rheumatology Antwerp University Hospital Belgium; ^59^ Faculty of Biology, Medicine and Health, Centre for Musculoskeletal Research The University of Manchester Manchester UK; ^60^ Clinical Dermatology, Department of Dermatology University of California San Francisco California USA; ^61^ Department of Immunology and Allergy University Hospital of Wales UK; ^62^ Division of Allergy, Immunology and Rheumatology – Department of Pediatrics Federal University of São Paulo Brazil; ^63^ Department of Dermatology, Urticaria Center of Reference and Excellence Clinic, Kayseri Faculty of Medicine, Kayseri City Hospital Health Sciences University Turkey; ^64^ Clinica Universidad de Navarra, Allergy Spain; ^65^ Dermatology Unit Fondazione IRCCS Ca' Granda Ospedale Maggiore Policlinico of Milan Milan Italy; ^66^ Department of Respiratory Medicine Immunology/Allergy Clinic, Box Hill Hospital Australia; ^67^ Moscow City Research and Practical Center of Allergology and Immunology, City Clinical Hospital 52 Russia; ^68^ 2. Dept. of Clinical Immunology and Allergology I.M. Sechenov First Moscow State Medical University (Sechenov University) Moscow Russian Federation; ^69^ Allergy and Immunology NYU Grossman Long Island School of Medicine USA; ^70^ Food and Drug Allergy Center, Allergy and Immunology Cairo Egypt; ^71^ Ymune, Allergy and Immunology Brazil; ^72^ Department of Dermatology Osaka Medical and Pharmaceutical University Japan; ^73^ Emedic Salud, San Isidro Lima Peru; ^74^ Fundación Santa fe de Bogotá‐ Faculty of medicine Universidad de los Andes‐ UNIMEQ ORL Colombia; ^75^ Department of Dermatology, Faculty of Medicine University of Debrecen Hungary; ^76^ Immunology and Allergic Diseases Istanbul Faculty of Medicine Istanbul University Turkey; ^77^ Department of Dermatology, The Second Affiliated Hospital School of Medicine, Xi'an Jiaotong University Xi'an China; ^78^ Dr. D Y Patil Medical College and Hospital India; ^79^ Dermatology, University Hospital, Coimbra Local Health Unit and Faculty of Medicine University of Coimbra Portugal; ^80^ David Tvildiani Medical University Tbilisi Georgia; ^81^ St John's Institute of Dermatology, Guy's Hospital London UK; ^82^ Department of Dermatology Vrije Universiteit Brussel (VUB) Belgium; ^83^ Universitair Ziekenhuis Brussel (UZ Brussel) Belgium; ^84^ Instituto Politécnico Nacional, SEPI/ENMH Mexico; ^85^ Hospital Universitari Vall d'Hebron Spain; ^86^ Department of Allergy Universitat Autònoma de Barcelona Spain; ^87^ aha! Allergiezentrum Schweiz Switzerland; ^88^ Pediatrics, Evangelisches Klinikum Bethel, Universitätsklinikum OWL, Universität Bielefeld Germany; ^89^ Oregon Medical Research Center, Portland Oregon USA; ^90^ Nihon University Itabashi Hospital Center for Allergy Japan; ^91^ Division of Evidence‐Based Medicine (dEBM), Charité – Universitätsmedizin Berlin Germany; ^92^ Hiroshima University Hospital Japan; ^93^ Hiroshima City Hiroshima Citizens Hospital Japan; ^94^ Department of Dermatology University Hospital Linz, Johannes Kepler University Austria; ^95^ Department of Dermatology Showa University School of Medicine Japan; ^96^ Seoul National University Hospital, Division of Allergy and Clinical Immunology, ACARE Republic of Korea; ^97^ Medical University of South Carolina USA; ^98^ Dermatology & Allergy Hannover Medical School Germany; ^99^ Allergy and Immunology Department American University of Beirut Medical Center Lebanon; ^100^ Department of Clinical Allergology and Urticaria of Medical University of Silesia Poland; ^101^ Immunology/Allergy Unit Campbelltown Hospital and Western Sydney University Sydney Australia; ^102^ Division of Allergy and Clinical Immunology, Bnai Zion Medical Center, Affiliated with Rappaport Faculty of Medicine Technion‐Israel Institute of Technology Haifa Israel; ^103^ Allergy Research Center Mashhad University of Medical Sciences Mashhad Iran; ^104^ Icahn School of Medicine at Mount Sinai Hospital, Kimberaly and Eric J. Waldman Department of Dermatology USA; ^105^ Department of Dermatology Koç University School of Medicine Istanbul Turkey; ^106^ Department of Internal Medicine, Asthma and Allergy Medical University of Lodz Poland; ^107^ Department of Allergy and Clinical Immunology 424 General Military Training Hospital Greece; ^108^ University Clinic of Respiratory and Allergic Diseases Golnik Slovenia; ^109^ Dermatology Venereology and Pediatric Dermatology Clinic Medical University of Lublin Poland; ^110^ Department of Dermatology, Faculty of Medicine Siriraj Hospital Siriraj UCARE Thailand; ^111^ Department of Dermatology PGIMER Urticaria Clinic Chandigarh India; ^112^ Clinica San Felipe, Allergy Department Peru; ^113^ Centro de Excelencia en Asma y Alergia Larenas Hospital Médica Sur Mexico; ^114^ 2nd Department of Dermatology, Papageorgiou General Hospital Aristotle University of Thessaloniki Greece; ^115^ Department of Medicine, School of Clinical Medicine The University of Hong Kong China; ^116^ Leader Research, Division of Dermatology McMaster University Hamilton Canada; ^117^ Department of Dermatology, Venereology and Allergology University Medical Center Goettingen Germany; ^118^ Allergy Unit, 2nd Dpt of Dermatology and Venereology, National and Kapodistrian University of Athens University General Hospital ‘Attikon’ Greece; ^119^ Immunology and Allergology Department Hospital de São Bernardo, ULS Arrábida Portugal; ^120^ Department of Pathophysiology and Transplantation Università degli Studi di Milano, Milan Italy; ^121^ Centro Medico Vitae, Allergy and Clinical Immunology Argentina; ^122^ Department of Clinical Immunology and Allergology Raisa Meshkova Smolensk State Medical University Russia; ^123^ Adult Allergy & Immunology Division, Department of Medicine Hamad Medical Corporation Qatar; ^124^ Department of Dermatology and Allergy Center Odense University Hospital Denmark; ^125^ University Medical Center Groningen the Netherlands; ^126^ Chest and Allergy Clinic Imperial College Healthcare NHS Trust London UK; ^127^ Dermatology McGill University Health Centre Montreal Canada; ^128^ Operative Unit of Allergy and Clinical Immunology – Regional Reference Center for Allergic and Immunological Diseases, Department of Precision and Regenerative Medicine and Ionian Area University of Bari Aldo Moro Bari Italy; ^129^ Instituto Nacional de Pediatria, Unidad de Genetica de la Nutricion Mexico; ^130^ Pontificia Universidad Católica de Chile, Department of Dermatology; DermaCDU, UCARE Center Chile; ^131^ University of Tartu Estonia; ^132^ Department of Dermatology, Faculdade de Medicina FMUSP Universidade de Sao Paulo Sao Paulo SP Brazil; ^133^ Department of Dermatology and Venereology, Istanbul Faculty of Medicine Istanbul University Istanbul Turkey; ^134^ Hospital Italiano de Buenos Aires Argentina; ^135^ Pediatric Nippon Medical School Japan; ^136^ Allergy and Immunology Unit, University of Cape Town Lung Institute, Division of Allergy and Clinical Immunology University of Cape Town South Africa; ^137^ University Hospital “Alexandrovska”, Clinic of Allergology Bulgaria; ^138^ Department of Biomedical, Surgical and Dental Sciences University of Milan Milan Italy; ^139^ Clinical Dermatology, IRCCS Istituto Ortopedico Galeazzi Milan Italy; ^140^ College of Medicine and Sagore Dutta Hospital Kolkata India; ^141^ University Hospital Sv. Ivan Rilski Bulgaria; ^142^ Center of Clinical and Environmental Allergology University Hospital in Krakow Poland; ^143^ Department of Clinical and Environmental Allergology Jagiellonian University Medical College Poland; ^144^ Instituto de Alergia e Inmunologia del sur, Alergia e Inmunologia Argentina; ^145^ 1st Department of Internal Medicine, Uniclinic, UNA Paraguay; ^146^ Allergy and Immunology Clinic, Division of Allergy and Immunology, Department of Pediatrics University of the Philippines – PGH Philippines; ^147^ East Tallinn Central Hospital, Center of Allergology and Immunology Estonia; ^148^ Clinical and Diagnostic Immunology, St. James's Hospital Dublin Ireland; ^149^ Allergy & Immunology, Department of Medicine University of California, San Diego USA; ^150^ Allergy Department Hospital Italiano de Buenos Aires Argentina; ^151^ Allergy and Immunology Federal University of Parana Brazil; ^152^ Allergy Department Centro Hospitalar Vila Nova De Gaia Portugal; ^153^ London Allergy and Immunology Centre, Adult and Paediatric Allergy UK; ^154^ Faculty of Medicine Geomedi University Tbilisi Georgia; ^155^ Urticaria Clinic, St John's Institute of Dermatology, Guy's and St Thomas' Hospital UK; ^156^ Department of Pediatric Allergy Hacettepe University Faculty of Medicine Children's Hospital Turkey; ^157^ Johns Hopkins Asthma and Allergy Center USA; ^158^ Al Kindi Specialized Hospital W.L.L Bahrain; ^159^ Department of Dermatology Acıbadem Mehmet Ali Aydınlar University School of Medicine Turkey; ^160^ Group of Clinical and Experimental Allergy, Hospital “Alma Mater de Antioquia” University of Antioquia Colombia; ^161^ Allergy Unit in Internal Department Rimini Infermi Hospital Italy; ^162^ Department of Dermatology University Hospital Jena Germany; ^163^ Department of Dermatology University Hospital of Zürich Medicine Campus Davos Switzerland; ^164^ UCARE Hospital Santa Casa de Misericórdia de Vitória, Internal Medicine Brazil; ^165^ Allergy & Immunology Chughtai Medical Center, Lalik Chowk, DHA 2 Pakistan; ^166^ Department of Allergy & Immunology Kaiser Permanente Los Angeles Medical Center USA; ^167^ Sheikh Khalifa City Hospital Abu Dhabi UAE; ^168^ Department of Pulmonology and Allergology, School of Medicine Mongolia‐Japan Hospital of Mongolian National University of Medical Sciences Mongolia; ^169^ Hopital Tenon, APHP, Médecine Sorbonne Université, Service de Dermatologie et Allegologie France; ^170^ CINTESIS@RISE, Centre for Health Technology and Services Research, Health Research Network, Faculty of Medicine University of Porto Porto Portugal; ^171^ MEDCIDS, Department of Community Medicine, Information and Health Decision Sciences, Faculty of Medicine University of Porto Porto Portugal; ^172^ Clinic of Allergology, University Hospital Alexandrovska, Department of Allergy Medical University – Sofia Bulgaria; ^173^ Department of Dermatology University Medical Center Mainz Germany; ^174^ Dermatology Section, Department of Medicine and Surgery University of Perugia Perugia Italy; ^175^ Department of Dermatology and Venerology Faculty of Medicine at the Bezmialem Vakif University Turkey; ^176^ Division of Clinical Immunology and Allergy University of Toronto Canada; ^177^ Department of Dermatology JA Hiroshima General Hospital Japan; ^178^ Department of Dermatology Hiroshima University Hospital Japan; ^179^ Dermatology Department Remedika General Hospital North Macedonia; ^180^ Department of Dermatology Bispebjerg Hospital Denmark; ^181^ Division of Allergy and Clinical Immunology Erciyes University School of Medicine Turkey; ^182^ Department of Allergology, Medical University of Sofia, Clinic of Allergology University Hospital “Alexandrovska” Sofia Bulgaria; ^183^ Clementino Fraga Filho University Hospital, Federal University of Rio de Janeiro, Brazil, Internal Medicine Brazil; ^184^ Department of Dermatology Erasmus MC the Netherlands; ^185^ Allergy Department Complexo Hospitalario Universitario A Coruña Spain; ^186^ Department of Dermatology Aarhus University Hospital Denmark; ^187^ Department of Dermatology & Allergy Hannover Medical School Germany; ^188^ Department of Dermatology Hospital Pakar Sultanah Fatimah Malaysia; ^189^ Shanghai Skin Disease Hospital Tongji University School of Medicine China; ^190^ Allergy and Clinical Immunology Ajou University Medical Center South Korea; ^191^ Clinical Immunology and Allergy Department Frimley UK; ^192^ Dr Philip Frost Department of Dermatology University of Miami Miller School of Medicine USA; ^193^ Psychodermatology Department Medical University of Lodz Poland; ^194^ Department of Dermatology Tianjin Institute of Integrative Dermatology, Tianjin Academy of Traditional Chinese Medicine Affiliated Hospital Tianjin China; ^195^ Hospital del Mar Research Institute, Universitat Pompeu Fabra Barcelona Spain

**Keywords:** angioedema, consensus, evidence‐based, hives, itch, mast cell, urticaria, wheals

## Abstract

This update and revision of the international guideline for urticaria was developed in accordance with the methods recommended by Cochrane and the Grading of Recommendations Assessment, Development and Evaluation (GRADE) working group. It is an initiative of the Global Allergy and Asthma Excellence Network (GA^2^LEN) and its Urticaria and Angioedema Centers of Reference and Excellence (UCAREs and ACAREs), with the participation of 210 delegates from 107 national and international societies, from 59 countries. The consensus conference was held on December 6th, 2024. This guideline was acknowledged and accepted by the European Union of Medical Specialists (UEMS). Urticaria is a frequent, mast cell‐driven disease, defined by a rapid appearance of wheals, angioedema, or both. The lifetime prevalence of acute urticaria is estimated to be approximately 20%. Chronic urticaria, categorized as either chronic spontaneous urticaria or chronic inducible urticaria, is disabling, impairs quality of life, and affects performance at work and school, however, novel therapies are available. This updated version of the international guideline for urticaria covers the definition and classification of urticaria and outlines expert‐guided and evidence‐based diagnostic and therapeutic approaches for the different subtypes of urticaria.

## Graphical abstract

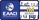

**Abbreviations**
AAS: angioedema activity score
ACARE: Angioedema Center of Reference and Excellence
ACE: angiotensin‐converting enzyme
AECT: angioedema control test
AE‐QoL: angioedema quality of life questionnaire
AGREE: Appraisal of Guidelines Research and Evaluation
AOSD: Adult‐onset Still's disease
APAAACI: Asia Pacific Association of Allergy, Asthma and Clinical Immunology
APST: Autologous Plasma Skin Test
ARIA: allergic rhinitis and its impact on asthma
ASST: autologous serum skin test
BAT: basophil activation test
BHRA: basophil histamine release assay
BSACI: British Society for Allergy & Clinical Immunology
C1INH: C1 esterase inhibitor
C4: C4 Complement Protein
CAPS: cryopyrin‐associated periodic symptoms
CholUAS: cholinergic urticaria activity score
CIndU: chronic inducible urticaria
CNS: central nervous system
COI: conflict of interest
COX‐2: cyclooxygenase‐2 enzyme
CRP: C‐reactive protein
CSU: chronic spontaneous urticaria
CSU^aiTI^
: chronic spontaneous urticaria autoimmunity Type I or autoallergic CSU
CSU^aiTIIb^
: chronic spontaneous urticaria autoimmunity Type IIb
CSU^uc^
: CSU due to an unknown cause
CU: chronic urticaria
CU‐Q2oL: Chronic urticaria quality of life questionnaire
CYP: cytochrome P
DPU: delayed pressure urticaria
EAACI: European Academy of Allergology and Clinical Immunology
EDF: European Dermatology Forum
EDHM: Eosinophilic Dermatosis of Hematologic Malignancy
ESR: erythrocyte sedimentation rate
EtD: evidence‐to‐decision
FCAS: Familial Cold Autoinflammatory Syndrome
FDA: [The United States] Food and Drug Administration
GA2CI: Gulf Academy of Allergy and Clinical Immunology
GA^2^LEN: Global Asthma and Allergy Excellence Network
GRADE: Grading of Recommendations Assessment, Development and Evaluation
HAE: hereditary angioedema
HES: hypereosinophilic syndromes
HIDS: hyper‐IgD syndrome
IgE/G: immunoglobulin E/G
IL‐4/13: interleukin 4/13
IVIG (also IGIV): intravenous immunoglobulins
MCAS: mast cell activation syndrome
MRGPRX2: Mas‐related G‐protein coupled receptor member X2
MWS: Muckle‐Wells‐Syndrome
NOMID: Neonatal Onset Multisystem Inflammatory Disease
N‐PF: non‐personal financial interest
NSAID: nonsteroidal anti‐inflammatory drugs
OAS: oral allergy syndrome
PAF: platelet activating factor
PET: positron emission tomography
PF: personal financial interest
PICO: Patient/Problem/Population, Intervention, Comparison/Control/Comparator, Outcome (a technique used in evidence‐based medicine)
PN‐F: personal nonfinancial interest
PROM: patient‐reported outcome measure
QoL: quality of life
REM: rapid eye movement
sJIA: systemic‐onset juvenile idiopathic arthritis
TNF: tumor necrosis factor
TPO: thyroid peroxidase
TRAPS: tumor necrosis factor receptor alpha‐associated periodic syndrome
UAS: urticaria activity score
UCARE: urticaria center of reference and excellence
UCT: urticaria control test
UEMS: European Union of Medical Specialists
UV: ultraviolet
WHO: World Health Organization

## 1 Introduction

The guideline is an initiative of the Global Allergy and Asthma Excellence Network (GA^2^LEN) and its Urticaria and Angioedema Centers of Reference and Excellence (UCAREs and ACAREs), the European Dermatology Forum (EDF), the Asia Pacific Association of Allergy, Asthma and Clinical Immunology (APAAACI), the American Academy of Dermatology (AAD), the British Society for Allergy & Clinical Immunology (BSACI), and the Gulf Academy of Allergy and Clinical Immunology (GA2CI), providing funding for the development of the guideline, which is an update and revision of the International EAACI/GA^2^LEN/EuroGuiDerm/APAAACI guideline on urticaria published in 2022 [1]. There was no funding from other sources, except that several of the supporting societies have provided travel funding for their delegates.

The present update and revision of the guideline was undertaken by a panel of 213 urticaria experts from 59 countries, nominated as delegates by 107 participating national and/or international medical or scientific societies and patient organizations (Table 1). All the aforementioned societies endorse this guideline. The update and revision of this guideline is based on evidence and expert consensus and was developed following the methods recommended by Cochrane and the Grading of Recommendations Assessment, Development and Evaluation (GRADE) working group. The work of the expert panel was supported by a team of EuroGuiDerm methodologists led by PD Dr. Ricardo Werner (Table 2) and included the contributions of the participants of the hybrid consensus conference, held on December 6th 2024, in Berlin, Germany.

**TABLE 1:** Members of the expert panel.

Title	First name	Last name	Country	Nominating society	Affiliations	Role
Dr.	Zainab	Abdul Hameed Ansari	Oman	Gulf Academy of Allergy and Clinical Immunology	Clinical Immunology & Allergy, Internal Medicine, Royal Hospital, Oman	Co‐author
Dr.	Amir Hamzah	Abdul Latiff	Malaysia	Malaysian Society of Allergy and Immunology	Pantai Hospital Kuala Lumpur, Allergy & Immunology Centre	Co‐author
Prof.	Mohamed	Abuzakouk	United Arab Emirates	Gulf Academy of Allergy and Clinical Immunology	Cleveland Clinic Abu Dhabi, Allergy and Immunology	Co‐author
Dr.	Maria Socorro	Agcaoili‐De Jesus	Philippines	Philippine Society of Allergy, Asthma & Immunology	University of the Philippines‐Philippine General Hospital, Division of Allergy and Immunology, Department of Medicine	Co‐author
Dr.	Rosana Câmara	Agondi	Brazil	Brazilian Association of Allergy and Clinical Immunology	Hospital das Clínicas da Universidade de São Paulo, Clinical Immunology and Allergy Division—Internal Medicine Department	Co‐author
Prof.	Mona	Al‐Ahmad	Kuwait	Kuwait Society of Allergy and Clinical Immunology	College of Medicine, Kuwait University, Kuwait, and Al‐Rashed Allergy Center, Ministry of Health, Kuwait	Co‐author
Prof.	Abdullah A.	Alangari	Saudi Arabia	Saudi Allergy Asthma and Immunology Society	Allergy and Immunology Unit, Department of Pediatrics, College of Medicine, King Saud University	Co‐author
Dr.	Hamad	Alhameli	United Arab Emirates	Gulf Academy of Allergy and Clinical Immunology	Cleveland Clinic Abu Dhabi, Allergy and Clinical immunology	Co‐author
Dr.	Cesar Daniel	Alonso Bello	México	Colegio mexicano de immunología clínica y alergia	Hospital Juárez de México, Allergy and Immunology	Co‐author
Dr.	Saad	Alshareef	Saudi Arabia	Gulf Academy of Allergy and Clinical Immunology	Allergy Immunology, King Faisal Specialist Hospital and Research Center	Co‐author
Dr.	Salem	Al‐Tamemi	Oman	Gulf Academy of Allergy and Clinical Immunology	Sultan Qaboos University Hospital, Child Health; Omani Society of Allergy & Clinical Immunology	Co‐author
Dr.	Sabine	Altrichter	Austria	Austrian society for Allergology and Immunology	Kepler University Hospital, Department of Dermatology and Venerology	Co‐author
M.D. MSc.	João Luís	Alves Marcelino	Portugal	Portuguese Society for Allergology and Clinical Immunology	Immunology and Allergology Department, Hospital de São Bernardo, ULS Arrábida, Portugal	Co‐author
Dr.	Humaid A.	Al Wahshi	Oman	Oman Society of Rheumatology (OSR)	Royal Hospital, Oman	Co‐author
Dr.	Susan	Aquilina	Malta	Maltese Association of Dermatology and Venereology	Department of Dermatology, Mater Dei Hospital, Malta	Co‐author
Dr.	Miriam	Araújo	Portugal	Sociedade Portuguesa de Alergologia e Imunologia Clinica	Serviço de Imunoalergologia do Hospital Dona Estefânia, CHULC E.P.E	Co‐author
Dr.	Rand	Arnaout	Saudi Arabia	Gulf Academy of Allergy and Clinical Immunology	King Faisal Specialist Hospital and Research Center, Medicine	Co‐author
Dr.	Riccardo	Asero	Italy	Associazione Allergologi Immunologi Italiani Territoriali e Ospedalieri	Clinica San Carlo, Ambulatorio di Allergologia	Co‐author
Prof.	Barbara	Ballmer‐Weber	Switzerland	Swiss Society of Allergy and Clinical Immunology	Department of Dermatology, University Hospital Zurich and Clinic for Dermatology and Allergology Kantonsspital St. Gallen	Co‐author
Dr.	Christine	Bangert	Austria	Austrian Society of Dermatology and Venereology	Medical University of Vienna, Department of Dermatology	Co‐author
Prof.	Andrea	Bauer	Germany	Arbeitsgemeinschaft für Berufs‐ und Umweltdermatologie e.V.	University Hospital Carl Gustav Carus, Department of Dermatology	Co‐author
Dr.	Moshe	Ben‐Shoshan	Canada	Canadian Society of Allergy and Clinical Immunology	Montreal Children's Hospital allergy clinic, Pediatrics, Division of Allergy Immunology and Dermatology	Co‐author
Dr.	Jonathan	Bernstein	USA	UCARE	University of Cincinnati Department of Internal Medicine, Division of Rheumatology, Allergy and Immunology, Advanced Allergy Services LLC and Bernstein Clinical Research Center LLC	Co‐author
Prof.	Carsten	Bindslev‐Jensen	Denmark	Danish Society of Allergology (DSA)	Allergy Center—ORCA, Department of Dermatology and Allergy Center—ORCA	Co‐author
Prof.	Mojca	Bizjak	Slovenia	Slovenian Association of Allergology & Clinical Immunology, Association of Slovenian Dermatovenereologists	University Clinic of Respiratory and Allergic Diseases Golnik, Division of Allergy; Faculty of Medicine, University of Ljubljana, Slovenia; Faculty of Medicine, University of Maribor, Slovenia	Co‐author
Dr.	Isabelle	Boccon‐Gibod	France	Société Française d'Allergologie (SFA)	National Reference Center for Angiœdema CREAK, Internal medicine and Clinical Immunology	Co‐author
Dr.	Hanna	Bonnekoh	Germany	Autoinflammation Network e.V.	Institute of Allergology, Charité—Universitätsmedizin Berlin, Corporate Member of Freie Universität Berlin and Humboldt‐Universität zu Berlin, Berlin, Germany; Fraunhofer Institute for Translational Medicine and Pharmacology ITMP, Immunology and Allergology, Berlin, Germany	Co‐author
Prof.	Laurence	Bouillet	France	CREAK‐Centre de reference for bradykinin‐mediated angioedema	CREAK, Service de médecine interne, CHUGA	Co‐author
Prof.	Knut	Brockow	Germany	German Dermatological Society	Department of Dermatology and Allergy Biederstein, Faculty of Medicine and Health, Technical University of Munich	Co‐author
Prof.	Zenon	Brzoza	Poland	Polish Society of Allergology	University of Opole, Department of Internal Diseases, Allergology, Endocrinology, and Gastroenterology	Co‐author
Dr.	Maja	Bulatović Ćalasan	The Netherlands	Dutch Society of Allergology and Clinical Immunology	University Medical Center Utrecht, Rheumatology and Clinical Immunology	Co‐author
Dr.	Adeeb	Bulkhi	Saudi Arabia	Saudi Allergy Asthma and Immunology Society	Department of Internal Medicine, College of Medicine, Umm Al‐Qura University, Makkah, Saudi Arabia	Co‐author
Dr.	Thomas	Buttgereit	Germany	ACARE	Institute of Allergology, Charité—Universitätsmedizin Berlin, Corporate Member of Freie Universität Berlin and Humboldt‐Universität zu Berlin, Berlin, Germany; Fraunhofer Institute for Translational Medicine and Pharmacology ITMP, Immunology and Allergology, Berlin, Germany	Co‐author
Prof.	Anette	Bygum	Denmark	Danish Dermatological Society	Clinical Institute, University of Southern Denmark	Co‐author
Dr.	Teresa	Caballero	Spain	The Spanish Society of Allergology and Clinical Immunology	Allergy Department, Hospital Universitario La Paz, IdiPAZ, CIBERER	Co‐author
Dr.	Oscar	Calderon	Peru	Sociedad Peruana de Alergia, Asma e Inmunología	Clinica Sanna el Golf, San Isidro, Allergy Unit	Co‐author
Prof.	Regis	Campos	Brazil	Brazilian Association of Allergy and Clinical Immunology	Complexo Hospitalar Universitário, Universidade Federal da Bahia, Internal Medicine	Co‐author
Dr.	Mauro	Cancian	Italy	Italian Network for Hereditary and Aquired Angioedema	Departmental Allergy Unit, Department of Systems Medicine	Co‐author
Ms.	Emily	Carne	United Kingdom	All Wales Allergy Network	Allergy Service for Wales	Co‐author
Prof.	Mary Anne	Castor	Philippines	Philippine Society of Allergy, Athma & Immunology	Division of Allergy and Immunology, Department of Pediatrics, University of the Philippines Manila ‐ Philippine General Hospital	Co‐author
Dr.	Inmaculada	Cerecedo	United Arab Emirates	Gulf Academy of Allergy and Clinical Immunology	Cleveland Clinic Abu Dhabi, Allergy and Clinical Immunology, Respiratoy Institute	Co‐author
Dr.	Tubanur	Çetinarslan	Turkey	Dermatovenereology Association of Türkiye	Manisa Celal Bayar University, Dermatology and Venereology	Co‐author
Prof.	Ivan	Cherrez‐Ojeda	Ecuador	Sociedad Ecuatoriana de Alergia, Asma e Inmunología Centro Sur Occidental	Espiritu Santo University; Institute of Allergology, Charité‐Universitätsmedizin Berlin, Corporate Member of Freie Universität Berlin and Humboldt University; Respiralab Research Center	Co‐author
Dr.	Nana	Chkhikvadze	Georgia	European Academy of Allergy and Clinical Immunology	Allergy and Immunology, Center of Allergy and Immunology, Tbilisi, Georgia	Co‐author
Prof.	Herberto Jose	Chong‐Neto	Brazil	Brazilian Society of Pediatrics	Serviço de Alergia e Imunologia, Complexo Hospital de Clínicas, Universidade Federal do Paraná, Pediatrics	Co‐author
Dr.	Karen	Choo	Singapore	Allergy and Clinical Immunology Society, Singapore	Urticaria Clinic, Singapore General Hospital, Department of Dermatology	Co‐author
Prof.	George	Christoff	Bulgaria	Bulgarian Alliance of CLinical and Translational Allergy	Medical Center Excelsior Ltd.	Co‐author
Prof.	Chia‐Yu	Chu	Taiwan	Taiwanese Dermatological Association	Immunology Clinic, Department of Dermatology, National Taiwan University Hospital	Co‐author
Prof.	Niall	Conlon	Ireland	Irish Association of Allergy and Immunology	St. James's Hospital, Department of Clinical and Diagnostic Immunology	Co‐author
Dr.	Célia	Costa	Portugal	Sociedade Portuguesa de Alergologia e Imunologia Clinica	Serviço de Imunoalergologia, Unidade Local de Saúde Santa Maria (ULSSM), Lisboa, Portugal	Co‐author
Prof.	Timothy	Craig	USA	The American College of Allergy, Asthma and Immunology	Pediatrics, Ob/Gyn and Biomedical sciences, Penn State University, Hershey, PA, USA; Senior Advisor Vinmec International Hospital, Times City, Hanoi Vietnam	Co‐author
Prof.	Paulo Ricardo	Criado	Brazil	Brazilian Society of Dermatology	Alergoskin Alergia e Dermatologia SS Ltda, and Centro Universitário Faculdade de Medicina do ABC, Dermatology	Co‐author
Dr.	Inna	Danilycheva	Russia	Russian Association of Allergologists and Clinical Immunologists	NRC Institute of Immunology FMBA of Russia, Allegology	Co‐author
Prof. Dr.	Razvigor	Darlenski	Bulgaria	European Dermatology Forum	Excelsior Med Center/Acibadem Cityclinic Tokuda hospital (inpatients), Department of dermatology and venereology	Co‐author
Dr.	Erika	De Arruda Chaves	Peru	Sociedad Peruana de Alergia Asma e Immunologia	Clinica Anglo Americana, Allergy and Inmunology	Co‐author
Prof.	Laurence	de Montjoye	Belgium	Belgian Urticaria Working Group/Royal Belgian Society of Dermatology and Venereology	Dermatology Department, Cliniques Universitaires Saint‐Luc, Université catholique de Louvain	Co‐author
Prof.	Marie‐Sylvie	Doutre	France	The French Society of Dermatology	Bordeaux University Hospital, Dermatology	Co‐author
Dr.	Aurélie	Du‐Thanh	France	The French Society of Dermatology	CHU de Montpellier, Dermatology	Co‐author
Prof.	Didier	Ebo	Belgium	Koninklijke Artsenvereniging van Antwerpen V.Z.W. Royal Medical Association of Antwerp	Antwerp University Hospital, Immunology Allergology Rheumatology	Co‐author
Dr.	Shuayb	Elkhalifa	United Arab Emirates	Gulf Academy of Allergy and Clinical Immunology	Allergy and Immunology Division, Cleveland Clinic Abu Dhabi, Abu Dhabi, United Arab Emirates; Faculty of Biology, Medicine and Health, Centre for Musculoskeletal Research, The University of Manchester, Manchester, United Kingdom	Co‐author
Dr.	Sarina	Elmariah	USA	American Academy of Dermatology	Clinical Dermatology Department of Dermatology, University of California, San Francisco, USA	Co‐author
Dr.	Tariq	El‐Shanawany	Wales	British Society for Allergy and Clinical Immunology	University Hospital of Wales, Department of Immunology and Allergy	Co‐author
Dr.	Luis Felipe	Ensina	Brazil	Brazilian Association of Allergy and Clinical Immunology	Federal University of São Paulo, Division of Allergy, Immunology and Rheumatology—Department of Pediatrics	Co‐author
Prof.	Ragıp	Ertaş	Turkey	Turkish Society of Dermatology	Health Sciences University, Kayseri Faculty of Medicine, Kayseri City Hospital, Department of Dermatology, Urticaria Center of Reference and Excellence Clinic.	Co‐author
Prof.	Roberta	Fachini Jardim Criado	Brazil	Rede Urticária Brasil	Ucare Faculdade De Medicina Do Abc (Abc Medical School), Department of Dermatology	Co‐author
Prof.	Marta	Ferrer	Spain	The Spanish Society of Allergology and Clinical Immunology	Clinica Universidad de Navarra, Allergy	Co‐author
Dr.	Silvia	Ferrucci	Italy	Società italiana di Dermatologia Allergologica, Professionale e Ambientale	Dermatology unit. Fondazione IRCCS Ca′ Granda Ospedale Maggiore Policlinico of Milan. Italy	Co‐author
Dr.	Jie Shen (Jason)	Fok	Australia	Australasian Society of Clinical Immunology and Allergy	Immunology/Allergy Clinic, Box Hill Hospital, Department of Respiratory Medicine	Co‐author
Dr.	Daria	Fomina	Russia	Russian Association of Allergologists and Clinical Immunologists	Moscow City Research and Practical Center of Allergology and Immunology, City Clinical Hospital 52, Russia; 2. Dept. of Clinical Immunology and Allergology, I.M. Sechenov First Moscow State Medical University (Sechenov University), Moscow, Russian Federation	Co‐author
Prof.	Luz	Fonacier	USA	American College of Allergy, Asthma and Immunology	NYU Grossman Long Island School of Medicine, Allergy and Immunology	Co‐author
Dr.	Ghada E.	Fouda	Egypt	UCARE	Food and Drug Allergy Center, Allergy and Immunology, Cairo, Egypt	Co‐author
Ms.	Isadora	Francescantonio	Brazil	Associação dos Magistrados Brasileiros/Brazilian Society of Allergy and Immunology	Ymune, Allergy and Immunology	Co‐author
Dr.	Atsushi	Fukunaga	Japan	The Japanese Society of Allergology	Osaka Medical and Pharmaceutical University, Department of Dermatology	Co‐author
Dr.	Cesar Alberto	Galvan Calle	Peru	Peruvian Immunology Society	Emedic Salud, San Isidro, Lima	Co‐author
Dr.	Elizabeth	Garcia	Colombia	Asociación Colombiana de Alergia, Asma e Inmunología	Fundación Santa fe de Bogotá‐ Faculty of medicine Universidad de los Andes‐ UNIMEQ ORL	Co‐author
Dr.	Krisztián	Gáspár	Hungary	Hungarian Dermatological Society	University of Debrecen, Faculty of Medicine, Department of Dermatology	Co‐author
Prof.	Asli	Gelincik	Turkey	Turkish National Society of Allergy and Clinical Immunology	Istanbul Faculty of Medicine Istanbul University, Immunology and Allergic Diseases	Co‐author
Prof.	Songmei	Geng	China	Chinese Dermatologist Association	Department of Dermatology, The Second Affiliated Hospital, School of Medicine, Xi'an Jiaotong University, Xi'an, China	Co‐author
Prof.	Ana Maria	Giménez‐Arnau	Spain	Academia Española De Dermatologia y Venereologia	Hospital del Mar Research Institute. Universitat Pompeu Fabra, Barcelona, Spain	Co‐author
Prof.	Kiran	Godse	India	Indian Association of Dermatologists, Venereologists and Leprologists	Dr. D Y Patil Medical College and Hospital	Co‐author
Prof.	Margarida	Gonçalo	Portugal	Sociedade Portuguesa de Dermatologia e Venereologia	Dermatology, University Hospital, Coimbra Local health Unit and Faculty of Medicine, University of Coimbra	Co‐author
Prof.	Maia	Gotua	Georgia	Georgian Academy of Allergy, Asthma, and Clinical Immunology	Center of Allergy and Immunology, Tbilisi, Georgia, UCARE; David Tvildiani Medical University, Tbilisi, Georgia	Co‐author
Prof.	Clive	Grattan	United Kingdom	British Society for Allergy & Clinical Immunology	St John's Institute of Dermatology, Guy's Hospital, London, UK	
Prof.	Martine	Grosber	Belgium	Belgian Urticaria Working Group/Royal Belgian Society of Dermatology and Venereology	Department of Dermatology, Vrije Universiteit Brussel (VUB), Universitair Ziekenhuis Brussel (UZ Brussel)	Co‐author
Prof.	Guillermo	Guidos Fogelbach	México	Colegio mexicano de immunología clínica y alergia	Instituto Politécnico Nacional, SEPI/ENMH	Co‐author
Prof.	Mar	Guilarte	Spain	Spanish Allergology and Clinical Immunology Society	Hospital Universitari Vall d'Hebron; Universitat Autònoma de Barcelona. Departmemnt of Allergy.	Co‐author
Ms.	Roxane	Guillod	Switzerland	aha! Allergiezentrum Schweiz	aha! Allergiezentrum Schweiz	Co‐author
Prof.	Eckard	Hamelmann	Germany	Deutsche Gesellschaft für Allergologie und klinische Immunologie e.V.	Evangelisches Klinikum Bethel, Universitätsklinikum OWL, Universität Bielefeld, Pediatrics	Co‐author
Dr.	Jason	Hawkes	USA	The American Academy of Dermatology	Oregon Medical Research Center, Portland, Oregon	Co‐author
Dr.	Koremasa	Hayama	Japan	The Japanese Dermatological Association	Nihon University Itabashi Hospital Center for Allergy	Co‐author
Prof.	Michihiro	Hide	Japan	The Japanese Society of Allergology	Hiroshima University Hospital, and Hiroshima City Hiroshima Citizens Hospital; Dermatology	Co‐author
Prof.	Wolfram	Hoetzenecker	Austria	Österreichische Gesellschaft für Allergologie & Immunologie	University Hospital Linz, Johannes Kepler University, Department of Dermatology	Co‐author
Prof.	Naoko	Inomata	Japan	The Japanese Dermatological Association	Showa University School of Medicine, Dept. of Dermatology	Co‐author
Prof.	Hye‐Ryun	Kang	Republic of Korea	Korean Academy of Asthma, Allergy and Clinical Immunology	Seoul National University Hospital, Division of Allergy and Clinical Immunology, ACARE	Co‐author
Dr.	Allen	Kaplan	USA	GA^2^LEN	Medical University of South Carolina, Medicine	Co‐author
Prof.	Alexander	Kapp	Germany	Deutsche Akademie für Allergologie und Umweltmedizin e.V.	Hannover Medical School, Dermatology & Allergy	Co‐author
Dr.	Marilyn	Karam	United Arab Emirates	Gulf Academy of Allergy and Clinical Immunology	American University of Beirut Medical Center, Allergy and Immunology department, Lebanon	Co‐author
Prof.	Alicja	Kasperska‐Zajac	Poland	Polish Society of Allergology	Department of Clinical Allergology and Urticaria of Medical University of Silesia, Poland	Co‐author
Prof.	Constance H.	Katelaris	Australia	Australasian Society of Clinical Immunology and Allergy	Immunology/Allergy Unit, Campbelltown Hospital and Western Sydney University Sydney Australia	Co‐author
Prof.	Aharon	Kessel	Israel	Israel Association of Allergy and Clinical Immunology	Bnai Zion Medical Center, Division of Allergy and Clinical Immunology, Division of Allergy and Clinical Immunology	Co‐author
Prof.	Maryam	Khoshkhui	Iran	Iranian Society of Asthma and Allergy	Allergy Research Center, Mashhad University of Medical Sciences, Mashhad, Iran	Co‐author
Prof.	Brian	Kim	USA	International Forum for the Study of Itch	Icahn School of Medicine at Mount Sinai Hospital, Kimberaly and Eric J. Waldman Department of Dermatology	Co‐author
Prof.	Tamar	Kinaciyan	Austria	Österreichische Gesellschaft für Dermatologie und Venerologie	Department of Dermatology, Medical University of Vienna, Vienna, Austria	Co‐author
Prof.	Emek	Kocatürk	Turkey	Turkish Society of Dermatology	Bahçeşehir University, Dermatology	Co‐author
Dr.	Marta	Kolacinska‐Flont	Poland	Polish Society of Allergology	Department of Internal Medicine, Asthma and Allergy, Medical University of Lodz, Poland	Co‐author
Dr.	Pavel	Kolkhir	Germany	Urticaria Network e.V.	Institute of Allergology, Charité—Universitätsmedizin Berlin, Corporate Member of Freie Universität Berlin and Humboldt‐Universität zu Berlin, Berlin, Germany; Fraunhofer Institute for Translational Medicine and Pharmacology ITMP, Immunology and Allergology, Berlin, Germany	Co‐author
Dr.	George N.	Konstantinou	Greece	The Hellenic Society of Allergology & Clinical Immunology	424 General Military Training Hospital, Department of Allergy and Clinical Immunology	Co‐author
Prof.	Mitja	Kosnik	Slovenia	Slovenian Association of Allergology & Clinical Immunology	University Clinic of Respiratory and Allergic Diseases, Golnik, Slovenia; Faculty of Medicine, University of Ljubljana, Slovenia	Co‐author
Prof.	Dorota	Krasowska	Poland	Polish Dermatological Society	Dermatology, Venereology and Pediatric Dermatology Clinic Medical University of Lublin, Poland, Department of Dermatology, Venereology and Pediatric Dermatology Medical University of Lublin, Poland	Co‐author
Prof.	Kanokvalai	Kulthanan	Thailand	Dermatological Society of Thailand	Siriraj UCARE, Department of Dermatology, Faculty of Medicine Siriraj Hospital	Co‐author
Prof.	Muthu Sendhil	Kumaran	India	Indian Association of Dermatologists, Venereologists and Leprologists	Urticaria Clinic, Department of Dermatology PGIMER Chandigarh	Co‐author
Dr.	Izabela	Kuprys‐Lipinska	Poland	Polish Society of Allergology	Department of Internal Medicine, Asthma and Allergy, Medical University of Lodz, Poland	Co‐author
Dr.	Moises	Labrador	Spain	Spanish Society of Allergology and Clinical Immunology	Hospital Vall d'Hebron, Barcelona, Spain.	Co‐author
Dr.	Jose Ignacio	Larco	Peru	Sociedad peruana de alergia asma e immunologia	Clinica San Felipe, Allergy Department	Co‐author
Dr.	Désirée	Larenas‐Linnemann	Mexico	Mexican College of Pediatricians Specialists in Clinical Immunology and Allergy	Centro de Excelencia en Asma y Alergia Larenas Hospital Médica Sur	Co‐author
Dr.	Elena	Latysheva	Russia	Russian Association of Allergologists and Clinical Immunologists	NRC Institute of Immunology FMBA, Russia	Co‐author
Prof.	Elisavet	Lazaridou	Greece	Hellenic Dermoscopy Society	2nd Department of Dermatology, Papageorgiou General Hospital, Aristotle University of Thessaloniki	Co‐author
Prof.	Raquel	L. Orfali	Brazil	Brazilian Society of Dermatology	Department of Dermatology, Faculdade de Medicina FMUSP, Universidade de Sao Paulo, Sao Paulo, SP, BR; UCARE.	Co‐author
Prof.	Philip Hei	Li	China	Hong Kong Institute of Allergy	Department of Medicine, School of Clinical Medicine, The University of Hong Kong	Co‐author
Prof.	Hermenio	Lima	Canada	Dermatology Association of Ontario	Leader Research, Dermatology	Co‐author
Dr.	Undine	Lippert	Germany	North German Dermatological Society	University Medical Center Goettingen, Department of Dermatology, Venereology and Allergology	Co‐author
Prof. Dr.	Markus	Magerl	Germany	ACARE	Institute of Allergology, Charité—Universitätsmedizin Berlin, Corporate Member of Freie Universität Berlin and Humboldt‐Universität zu Berlin, Berlin, Germany; Fraunhofer Institute for Translational Medicine and Pharmacology ITMP, Immunology and Allergology, Berlin, Germany	Co‐author
Dr.	Michael	Makris	Greece	The Hellenic Society of Allergology & Clinical Immunology	Allergy Unit; 2nd Dpt of Dermatology and Venereology, National and Kapodistrian University of Athens, University General Hospital ‘Attikon’	Co‐author
Prof.	Angelo Valerio	Marzano	Italy	Società Italiana di Dermatologia medica, chirurgica, estetica e di Malattie Sessualmente Trasmesse (SIDeMaST)	Dermatology Unit, Fondazione IRCCS Ca′ Granda Ospedale Maggiore Policlinico, Milan, Italy; Department of Pathophysiology and Transplantation, Università degli Studi di Milano, Milan, Italy	Co‐author
Dr.	Iris	Medina	Argentina	UCARE	Centro Medico Vitae, Allergy and Clinical Immunology	Co‐author
Prof.	Raisa	Meshkova	Russia	Russian Association of Allergologists and Clinical Immunologists	Raisa Meshkova Smolensk State Medical University, Raisa Meshkova Department of clinical immunology and allergology	Co‐author
Prof.	Martin	Metz	Germany	UCARE	Institute of Allergology, Charité—Universitätsmedizin Berlin, Corporate Member of Freie Universität Berlin and Humboldt‐Universität zu Berlin, Berlin, Germany; Fraunhofer Institute for Translational Medicine and Pharmacology ITMP, Immunology and Allergology, Berlin, Germany	Co‐author
Dr.	Daniel	Micallef	Malta	Maltese Association of Dermatology and Venereology	Mater Dei Hospital, Department of Dermatology	Co‐author
Dr.	Ramzy	Mohammed Ali	Qatar	Qatar Allergy & Immunology society & Gulf Academy of Allergy and Clinical Immunology	Adult Allergy & Immunology Division, Department of Medicine, Hamad Medical Corporation	Co‐author
Prof.	Charlotte	Mortz	Denmark	Danish Society of Allergology (DSA)	Department of Dermatology and Allergy Center, Odense University Hospital, Denmark	Co‐author
Dr.	Melba	Munoz	Germany	UCARE	Institute of Allergology, Charité—Universitätsmedizin Berlin, Corporate Member of Freie Universität Berlin and Humboldt‐Universität zu Berlin, Berlin, Germany; Fraunhofer Institute for Translational Medicine and Pharmacology ITMP, Immunology and Allergology, Berlin, Germany	Co‐author
Dr.	Hanneke	N.G. Oude Elberink	Netherlands	Dutch Society of Allergology and Clinical Immunology	University Medical Center Groningen	Co‐author
Prof.	Alla	Nakonechna	United Kingdom	British Society for Allergy and Clinical Immunology	Imperial College Healthcare NHS Trust, Chest and Allergy Clinic, London, United Kingdom	Co‐author
Dr.	Iman	Nasr	Oman	Oman Society of Allergy & Clinical Immunology	Clinical Immunology and Allergy, Royal Hospital, Oman	Co‐author
Dr.	Elena	Netchiporouk	Canada	UCARE	McGill University Health Centre, Dermatology	Co‐author
Prof.	Eustachio	Nettis	Italy	Società Italiana di Allergologia, Asma e Immunologia Clinica	Operative Unit of Allergy and Clinical Immunology ‐ Regional Reference Center for Allergic and Immunological Diseases, Department of Precision and Regenerative Medicine and Ionian Area, University of Bari Aldo Moro, Bari, Italy	Co‐author
Dr.	Sandra	Nieto	Mexico	Asociación Mexicana de Angioedema Hereditario, A.C.	Instituto Nacional de Pediatria, Unidad de Genetica de la Nutricion	Co‐author
Dr.	Isabel	Ogueta C.	Chile	Chilean Society of Dermatology and Venereology, SOCHIDERM	Pontificia Universidad Católica de Chile, Department of Dermatology; DermaCDU, UCARE Center	Co‐author
Dr.	Tiia‐Linda	Okas	Estonia	Estonian Society of Dermatovenerologists	University of Tartu	Co‐author
Prof.	Esen	Özkaya	Turkey	Turkish Society of Dermatology	Istanbul Faculty of Medicine, Istanbul University, Istanbul, Turkey, Department of Dermatology and Venereology	Co‐author
Prof.	Claudio	Parisi	Argentina	UCARE	Hospital Italiano de Buenos Aires, Argentina	Co‐author
Prof.	Ruby	Pawankar	Japan	Asia Pacific Association of Allergy, Asthma and Clinical Immunology	Pediatric, Nippon Medical School	Co‐author
Dr.	Manuel P.	Pereira	Germany	UCARE	Institute of Allergology, Charité—Universitätsmedizin Berlin, Corporate Member of Freie Universität Berlin and Humboldt‐Universität zu Berlin, Berlin, Germany; Fraunhofer Institute for Translational Medicine and Pharmacology ITMP, Immunology and Allergology, Berlin, Germany	Co‐author
Prof.	Jonny	Peter	South Africa	Allergy Society of South Africa	Allergy and Immunology Unit, University of Cape Town Lung Institute, Division of Allergy and Clinical Immunology, University of Cape Town	Co‐author
Dr.	Elena	Petkova	Bulgaria	Bulgarian Society of Allergology	University Hospital “Alexandrovska”, Clinic of Allergology	Co‐author
Prof.	Paolo Daniele	Pigatto	Italy	Società Italiana di Dermatologia medica, chirurgica, estetica e di Malattie Sessualmente Trasmesse (SIDeMaST)	Department of Biomedical, Surgical and Dental Sciences, University of Milan, Milan, Italy; Clinical Dermatology, IRCCS Istituto Ortopedico Galeazzi, Milan, Italy	Co‐author
Dr.	Indrashis	Podder	India	Kolkata Derma Forum	College of Medicine and Sagore Dutta Hospital, Kolkata, India	Co‐author
Prof.	Todor	Popov	Bulgaria	Bulgarian Alliance of Clinical and Translational Allergy	University Hospital Sv. Ivan Rilski	Co‐author
Dr.	Grzegorz	Porebski	Poland	Polish Society of Allergology	Center of Clinical and Environmental Allergology, University Hospital in Krakow, Department of Clinical and Environmental Allergology, Jagiellonian University Medical College	Co‐author
Dr.	Polina	Pyatilova	Germany	Treasurer Urtikaria Network e.V.	Institute of Allergology, Charité—Universitätsmedizin Berlin, Corporate Member of Freie Universität Berlin and Humboldt‐Universität zu Berlin, Berlin, Germany; Fraunhofer Institute for Translational Medicine and Pharmacology ITMP, Immunology and Allergology, Berlin, Germany	Co‐author
Dr.	German Dario	Ramon	Argentina	GA^2^LEN	Instituto de Alergia e Inmunologia del sur, Alergia e Inmunologia	Co‐author
Prof. Dr.	Héctor Aurelio Ramón	Ratti Sisa	Paraguay	Sociedad Paraguaya de Alergia, Asma e Inmunologia	1st Department of Internal Medicine, Uniclinic, UNA	Co‐author
Prof.	Marysia	Recto	Philippines	Philippine Society of Allergy, Athma & Immunology	Allergy and Immunology clinic, Division of allergy and immunology, Department of Pediatrics, University of the Philippines – PGH	Co‐author
Dr.	Krista	Ress	Estonia	Estonian Society for Immunology and Allergology	East Tallinn Central Hospital, Center of Allergology and Immunology	Co‐author
Dr.	Katie	Ridge	Ireland	To be added	St. James's Hospital, Dublin, Ireland, Clinical and diagnostic Immunology	Co‐author
Dr.	Marc	Riedl	USA	Western Society of Allergy, Asthma & Immunology	University of California, San Diego, Allergy & Immunology, Department of Medicine	Co‐author
Dr.	Carla	Ritchie	Argentina	Argentinean Association of Allergy & Clinical Immunology	Allergy department, Hospital Italiano de Buenos Aires	Co‐author
Prof.	Nelson	Rosario Filho	Brazil	Associação Brasileira de Alergia e Imunologia	Federal University of Parana, Allergy and Immunology	Co‐author
Dr.	Isabel	Rosmaninho	Portugal	Sociedade Portuguesa de Alergologia e Imunologia Clinica	Allergy Department, Centro Hospitalar Vila Nova De Gaia	Co‐author
Prof.	Michael	Rudenko	United Kingdom	British Society for Allergy and Clinical Immunology	London Allergy and Immunology Centre, Adult and Pediatric Allergy	Co‐author
Prof.	Maia	Rukhadze	Georgia	Asthma and Allergy Patient's Asscoiation of Georgia	Center of Allergy&Immunology, Tbilisi, Georgia, Geomedi University, Faculty of Medicine, Tbilisi, Georgia	Co‐author
Dr.	Krzysztof	Rutkowski	United Kingdom	British Society for Allergy and Clinical Immunology	Urticaria Clinic, St John's Institute of Dermatology, Guy's and St Thomas' Hospital	Co‐author
Prof.	Vito	Sabato	Belgium	Koninklijke Artsenvereniging van Antwerpen V.Z.W. Royal Medical Association of Antwerp	Antwerp University Hospital, Immunology Allergology Rheumatology	Co‐author
Prof. Dr.	Umit Murat	Sahiner	Turkey	Turkish National Society of Allergy and Clinical Immunology	Hacettepe University Faculty of Medicine Children's Hospital, Department of Pediatric Allergy	Co‐author
Dr.	Sarbjit	Saini	USA	UCARE	Johns Hopkins Asthma and Allergy Center, Medicine	Co‐author
Dr.	Fadhel	Saleh Al Sabbagh	Bahrain	Gulf Academy of Allergy and Clinical Immunology	Al Kindi Specialized Hospital W.L.L	Co‐author
Dr.	Andaç	Salman	Turkey	Society of Dermatology and Venereology (Turkiye)	Acıbadem Mehmet Ali Aydınlar University School of Medicine, Department of Dermatology	Co‐author
Dr.	Fulvio	Salvo	United Arab Emirates	Gulf Academy of Allergy and Clinical Immunology	Cleveland clinic Abu Dhabi, Allergy and Clinical Immunology	Co‐author
Dr.	Jorge	Sanchez	Colombia	Asociación Colombiana de Alergia, Asma e Inmunología	Group of Clinical and Experimental Allergy, Hospital “Alma Mater de Antioquia”, University of Antioquia	Co‐author
Dr.	Annalisa	Santucci	Italy	Italian Association of Allergists and Immunologists	Rimini Infermi Hospital, Allergy unit in internal department	Co‐author
Dr.	Sibylle	Schliemann	Germany	Arbeitsgemeinschaft für Berufs‐ und Umweltdermatologie e.V.	University Hospital Jena, Department of Dermatology	Co‐author
Prof.	Peter	Schmid‐Grendelmeier	Switzerland	CK‐CARE	Dept. of Dermatology, University Hospital of Zürich Medicine Campus Davos	Co‐author
Prof.	Bulent Enis	Sekerel	Turkey	Turkish National Society of Allergy and Clinical Immunology	Hacettepe University School of Medicine, Department of Pediatric Allergy and Asthma, Ankara, Turkey	Co‐author
Dr.	Faradiba	Serpa	Brazil	Brazilian Association of Allergy and Clinical Immunology	UCARE Hospital Santa Casa de Misericórdia de Vitória, Internal Medicine	Co‐author
Dr.	Farrukh	Sheikh	Pakistan	Society of Rhinology & Endoscopic Skull Base Surgery	Chughtai Medical Center, Lalik Chowk, DHA 2, Allergy & Immunology	Co‐author
Dr.	Javed	Sheikh	USA	California Society of Allergy, Asthma and Immunology	Kaiser Permanente Los Angeles Medical Center, Department of Allergy & Immunology	Co‐author
Dr.	Hiba	Shendi	United Arab Emirates	Gulf Academy of Allergy and Clinical Immunology	Sheikh Khalifa City Hospital, Abu Dhabi	Co‐author
Dr.	Frank	Siebenhaar	Germany	European Mast cell and Basophil Research Network	Institute of Allergology, Charité—Universitätsmedizin Berlin, Corporate Member of Freie Universität Berlin and Humboldt‐Universität zu Berlin, Berlin, Germany; Fraunhofer Institute for Translational Medicine and Pharmacology ITMP, Immunology and Allergology, Berlin, Germany	Co‐author
Prof.	Munkhbayarlakh	Sonomjamts	Mongolia	Mongolian Society of Allergology	Mongolia‐Japan Hospital of Mongolian National University of Medical Sciences, Department of Pulmonology and Allergology, School of Medicine	Co‐author
Prof.	Angele	Soria	France	The French Society of Dermatology	Hopital Tenon, APHP, Médecine Sorbonne Université, Service de dermatologie et allegologie	Co‐author
Prof.	Maria	Staevska	Bulgaria	Bulgarian Society of Allergology	Clinic of Allergology, University Hospital Alexandrovska, Department of Allergy, Medical University ‐ Sofia	Co‐author
Prof.	Petra	Staubach	Germany	Urticaria Network e.V.	University Medical Center Mainz, Department of Dermatology	Co‐author
Prof.	Luca	Stingeni	Italy	chirurgica, estetica e di Malattie Sessualmente Trasmesse (SIDeMaST)	Dermatology Section, Department of Medicine and Surgery, University of Perugia, Perugia, Italy	Co‐author
Dr.	Marcin	Stobiecki	Poland	Polish Society of Allergology	Jagiellonian University Medical College, Department of Clinical and Environmental Allergology	Co‐author
Prof. Dr.	Özlem	Su Küçük	Turkey	Anatolian Dermatology and Cosmetology Society	Department of Dermatology and Venerology, Faculty of Medicine at the Bezmialem Vakif University	Co‐author
Prof.	Gordon	Sussman	Canada	Canadian Society of Allergy and Clinical Immunology	Division of Clinical Immunology and Allergy, University of Toronto, Canada.	Co‐author
Prof.	Andrea	Szegedi	Hungary	Hungarian Dermatological Society	University of Debrecen, Faculty of Medicine, Department of Dermatology	Co‐author
Dr.	Shunsuke	Takahagi	Japan	The Japanese Dermatological Association	JA Hiroshima General Hospital, Department of Dermatology	Co‐author
Prof.	Akio	Tanaka	Japan	The Japanese Society of Allergology	Hiroshima University Hospital, Department of Dermatology	Co‐author
Dr.	Natasa	Teovska Mitrevska	North Macedonia	Macedonian Dermatovenereologic Society	Remedika General Hospital, Dermatology Department	Co‐author
Prof.	Simon Francis	Thomsen	Denmark	Danish Dermatological Society	Bispebjerg Hospital, Department of Dermatology	Co‐author
Prof.	Elias	Toubi	Israel	Israel Association of Allergy and Clinical Immunology	Allergy and Clinical Immunology	Co‐author
Dr.	Fragkiski	Tsatsou	Greece	Hellenic Dermoscopy Society	2nd Department of Dermatology, Papageorgiou General Hospital, Aristotle University of Thessaloniki	Co‐author
Dr.	Murat	Turk	Turkey	Turkish National Society of Allergy and Clinical Immunology	Erciyes University School of Medicine, Division of Allergy and Clinical Immunology	Co‐author
Prof.	Zahava	Vadasz	Israel	Israel Association of Allergy and Clinical Immunology	Allergy and Clinical Immunology, Bnai‐Zion Medical Center, Haifa, Israel	Co‐author
Dr.	Anna	Valerieva	Bulgaria	Bulgarian Society of Allergology	Clinic of Allergology, Department of Allergology	Co‐author
Prof.	Solange	Valle	Brazil	Brazilian Association of Allergy and Clinical Immunology	Clementino Fraga Filho University Hospital, Federal University of Rio de Janeiro, Brazil, Internal Medicine	Co‐author
Dr.	Martijn	van Doorn	The Netherlands	De Nederlandse Vereniging voor Dermatologie en Venereologie	Erasmus MC, Department of Dermatology	Co‐author
Dr.	Beatriz	Veleiro Perez	Spain	The Spanish Society of Allergology and Clinical Immunology	Complexo Hospitalario Universitario A Coruña, Allergy Department	Co‐author
Dr.	Carolina Elisa	Vera Ayala	Germany	ACARE	Institute of Allergology, Charité—Universitätsmedizin Berlin, Corporate Member of Freie Universität Berlin and Humboldt‐Universität zu Berlin, Berlin, Germany; Fraunhofer Institute for Translational Medicine and Pharmacology ITMP, Immunology and Allergology, Berlin, Germany	Co‐author
Prof.	Christian	Vestergaard	Denmark	Danish Dermatological Society	Aarhus University Hospital, Department of Dermatology	Co‐author
Prof.	Celina	W. Maruta	Brazil	Brazilian Society of Dermatology	Department of Dermatology, Faculdade de Medicina FMUSP, Universidade de Sao Paulo, Sao Paulo, SP, BR; UCARE	Co‐author
Prof.	Bettina	Wedi	Germany	German Society for Allergology and Clinical Immunology	Hannover Medical School, Dept. of Dermatology & Allergy	Co‐author
Dr.	Evelyn Wen Yee	Yap	Malaysia	Dermatology Chapter, College of Physicians, Academy of Medicine of Malaysia	Hospital Pakar Sultanah Fatimah, Department of Dermatology	Co‐author
Prof.	Paraskevi	Xepapadaki	Greece	National and Kapodistrian University of Athens	2nd Pediatric Clinic, National and Kapodistrian University of Athens, Allergy Department	Co‐author
Dr.	Yi‐Kui	Xiang	China	Chinese Association of Rehabilitation Dermatology	Shanghai Skin Disease Hospital, Tongji University School of Medicine	Co‐author
Prof.	Young‐Min	Ye	South Korea	Korean Academy of Asthma, Allergy and Clinical Immunology	Ajou University Medical Center, Allergy and Clinical Immunology	Co‐author
Dr.	Patrick FK	Yong	UK	British Society for Allergy and Clinical Immunology	Frimley Park Hospital, Clinical Immunology and Allergy Department	Co‐author
Prof.	Gil	Yosipovitch	USA	International Forum for the Study of Itch	Dr Philip Frost Department of Dermatology University of Miami Miller School of Medicine	Co‐author
Prof.	Anna	Zalewska‐Janowska	Poland	Polish Society of Allergology	Psychodermatology Dpt. Medical University of Lodz	Co‐author
Prof. Dr.	Zuotao	Zhao	China	China Dermatologist Association, Chinese Society of Dermatology	Department of Dermatology, Tianjin Institute of Integrative Dermatology, Tianjin Academy of Traditional Chinese Medicine Affiliated Hospital Tianjin, China	Co‐author
Prof. Dr.	Torsten	Zuberbier	Germany	Global Asthma & Allergy Excellence Network	Institute of Allergology, Charité—Universitätsmedizin Berlin, Corporate Member of Freie Universität Berlin and Humboldt‐Universität zu Berlin, Berlin, Germany; Fraunhofer Institute for Translational Medicine and Pharmacology ITMP, Immunology and Allergology, Berlin, Germany	Co‐author

**TABLE 2:** Members of the EuroGuiDerm guideline methodology group.

Title	First name	Last name	Country	Organization	Role
	Martin	Dittmann	Germany	Division of Evidence‐Based Medicine (dEBM), Charité – Universitätsmedizin Berlin	Information specialist, team support
	Ruben	Heuer	Germany	Division of Evidence‐Based Medicine (dEBM), Charité – Universitätsmedizin Berlin	Methodologist
Dr.	Antonia	Pennitz	Germany	Division of Evidence‐Based Medicine (dEBM), Charité – Universitätsmedizin Berlin	Methodologist
	Christoph	Zeyen	Germany	Division of Evidence‐Based Medicine (dEBM), Charité – Universitätsmedizin Berlin	Methodologist
Dr.	Ricardo N.	Werner	Germany	Division of Evidence‐Based Medicine (dEBM), Charité – Universitätsmedizin Berlin	Senior methodologist, conference facilitator
Prof Dr.	Alexander	Nast	Germany	Division of Evidence‐Based Medicine (dEBM), Charité – Universitätsmedizin Berlin	Senior methodologist

The aim of the guideline is to provide a definition and classification of urticaria, thereby facilitating the interpretation of data from different centers and areas of the world with regard to underlying causes, eliciting factors, comorbidities, the burden on patients and society, and the therapeutic responsiveness of subtypes of urticaria. Furthermore, the guideline provides recommendations for diagnostic and therapeutic approaches in common subtypes of urticaria. This is an international guideline, with consideration given to the global diversity of patients, physicians, medical systems, and access to diagnosis and treatment.

## 2 Methods

The detailed methods used in the development of this guideline have been published as a separate Methods Report, which is available on the EDF website alongside a separate Evidence Report that includes all evidence‐to‐decision frameworks (https://www.guidelines.edf.one/edf‐guidelines‐and‐consensus‐statements).

The guideline takes into account the Appraisal of Guidelines Research and Evaluation (AGREE II) Instrument [2] and the methods suggested by the GRADE working group. The literature review was conducted using the methods given in the Cochrane Handbook for Systematic Reviews of Interventions [3].

In summary, experts from 106 societies were nominated to be involved in the development of this update and revision of the guideline. All members of the expert panel received an invitation to submit a declaration of their conflicts of interest (COIs) online and to self‐declare their personal financial interests (PF), nonpersonal financial interests (N‐PF), and personal nonfinancial interests (PN‐F). An overview of the declarations of P‐F conflicts of interest is given in the Methods Report. Overall, 124 members of the expert panel (58.21%) declared that they had no PF COIs.

In the 2025 update of the guideline, five new questions pertaining to novel treatments were introduced as well as a question regarding the use of biomarkers to predict the disease course. Aside from these alterations, the same key questions were used as those developed for the version of the guideline published in 2021 [1]. Details on the processes used to develop these questions are available in the Methods Report of the current guideline. The key questions were translated into the PICO format, which specifies the intervention, comparison and outcome used to assess efficacy and safety, and are included in the header of each evidence‐to‐decision framework. Systematic searches for randomized‐ and clinical controlled trials were conducted in three databases on 09 February 2024.

The search identified a total of 1946 records. Two independent reviewers evaluated the literature and extracted eligible data. The removal of duplicates and title/abstract screening resulted in 258 records to be assessed as full texts for eligibility, of which 246 were excluded. A total of 12 records were determined to fulfill the inclusion criteria. A graphical breakdown of this process and a list of excluded full‐text publications with reasons for exclusion can be found in the separate Methods Report.

Wherever possible, we calculated effect measures with confidence intervals and performed meta‐analyses using Review Manager [4]. The quality of the evidence was then assessed following the GRADE approach [5]. Five criteria (risk of bias, inconsistency, indirectness, imprecision, and publication bias) were evaluated for each outcome resulting in an overall assessment of quality of evidence (Table 3). Effect measures, such as risk ratios, were used to express the size of an effect, while the quality rating indicates the confidence that can be attributed to a result.

**TABLE 3:** Summary of the GRADE approach to assessing the quality of evidence by outcome in randomized controlled trials [7].

Initial rating of quality of the body of evidence	Criteria that may decrease the quality rating	Criteria that may increase the quality rating	Quality of the body of evidence
RCT: High NRSI: Low	– Risk of bias – Inconsistency – Indirectness – Imprecision – Publication bias	– Large effect – Dose response – Residual confounding	High (++++): We are very confident that the true effect lies close to that of the estimate of effect Moderate (+++): We are moderately confident in the effect estimate: The true effect is likely to be close to the estimate of the effect, but there is a possibility that it is substantially different Low (++): Our confidence in the effect estimate is limited: The true effect may be substantially different from the estimate of the effect Very low (+): We have very little confidence in the effect estimate: The true effect is likely to be substantially different from the estimate of effect

Abbreviations: NRSI, nonrandomized study of intervention (not included in this review as per inclusion criteria); RCT, randomized controlled trial.

Subsequently, evidence‐to‐decision frameworks were created to assist the expert panel in making judgments for specific comparisons regarding the magnitude of the desirable and undesirable effects, as the balance between these, and to provide an overview of the quality of the evidence. The evidence assessment yielded 12 new or updated GRADE evidence profiles and 12 new or updated evidence‐to‐decision frameworks. A summary of the evidence is provided in the separate Evidence Report. Recommendations for each of the evidence‐based key questions were subsequently drafted using standardized wording (Table 4).

**TABLE 4:** Standardized wording and symbols for guideline recommendations.

Strength of recommendation	Wording	Symbols	Implications
**Strong** recommendation **for** the use of an intervention	‘We **recommend** …’	**↑↑**	We believe that all or almost all informed people would make a choice in favor of using this intervention. Clinicians will not have to spend as much time on the process of decision‐making with the patient and may devote that time instead to overcoming barriers to implementation and adherence. In most clinical situations, the recommendation can be adopted as a policy
**Weak** recommendation **for** the use of an intervention	“We **suggest** …”	**↑**	We believe that most informed people would make a choice in favor of using this intervention, but a substantial number would not. Clinicians and other health care providers will need to devote more time to the process of shared decision‐making. Policy makers will have to involve many stakeholders and policy making will require substantial debate
**No recommendation** with respect to an intervention	“We **cannot make a recommendation** with respect to…”	**0**	Currently, a recommendation in favor of or against using this intervention cannot be made due to certain circumstances (e.g., unclear or balanced benefit–risk ratio, no data available)
**Weak** recommendation **against** the use of an intervention	“We **suggest against**…”	**↓**	We believe that most informed people would make a choice against using this intervention, but a substantial number would not
**Strong** recommendation **against** the use of an intervention	“We **recommend against**…”	**↓↓**	We believe that all or almost all informed people would make a choice against using this intervention. This recommendation can be adopted as a policy in most clinical situations

Prior to the consensus conference, a discussion and prevoting process was conducted via an online survey to familiarize the expert panel with the draft recommendations and evidence‐to‐decision frameworks. The survey also sought to gather feedback on these recommendations, and subsequently to use this feedback to modify the recommendations or to draft a series of alternative recommendations, to be presented and voted upon during the consensus conference. All members of the expert panel were eligible for discussion and prevoting process (regardless of whether they had P‐F conflicts of interests). Of the 213 members of the expert panel, 127 completed the prevoting survey (response rate of 59.62%). The results were subsequently provided to the expert panel for review or integrated into the evidence‐to‐decision frameworks. All evidence‐to‐decision frameworks and draft recommendations were made available in advance to the participants of the consensus conference.

The consensus conference was held on December 6th 2024 in a hybrid format. The participants consisted of the members of the expert panel and a broader group of over 100 professionals, including physicians regularly involved in treating patients with urticaria, basic or clinical researchers in the field, and representatives of patient organizations and advocacy groups. The conference was also open to the participation of industrial stakeholders, who, however, were ineligible to vote on the recommendations. Voting took place online using the Vevox polling platform. To be eligible to vote, participants were required to submit a conflict of interest (COI) declaration. All participants, except those who had a relevant COI or are employed at a pharmaceutical company, were eligible to vote and were provided with a code to access the live polls. During the conference, the nominal group technique was used to facilitate discussion, modification, and the attainment of agreement on the different recommendations [6]. Each draft recommendation was presented alongside the relevant evidence or justification; this was followed by an open discussion, preliminary voting, or collection of suggestions for alternative wording, and then the final vote. In accordance with the EuroGuiDerm Guideline Manual, strong consensus was defined as more than 95% agreement, whereas consensus was defined as 75%–95% agreement. All recommendations were voted on by at least 96 participants and were passed with at least 75% agreement.

After the conference, the text of the previously published guideline [1] was amended by the guideline coordinators and the methodologist team, in line with the voting results and the discussion during the preconference rounds of online voting and the conference. The draft was subsequently reviewed internally by the expert panel and externally by the participating national and international societies.

In the guideline, the strength of the consensus reached for each recommendation is reported as shown in Table 5.

**TABLE 5:** Definitions of strength of consensus according to EuroGuiDerm Guideline Manual.

**Strong consensus**	Agreement of > 95% participants
**Consensus**	Agreement of > 75%–95% participants
**Agreement of the majority**	Agreement of > 50%–75% participants

Each recommendation in the guideline is formatted as shown in Boxes 1, 2, 3. At the top of each box, the question of interest is given (e.g., “Should we … in chronic urticaria?”). In the row below the question of interest, the recommendation is stated using the standardized wording and symbols as shown in Table 4. In Box 1, for example, we can see that a strong recommendation is made (i.e., “We recommend…” and “↑↑” in dark green). Additionally, we can see, based on the information given in the right column of this row, that the eligible participants in the consensus conference agreed upon this recommendation and its wording with strong consensus (≥ 95% agreement), as well as that the recommendation is based on an expert consensus. If the recommendation is further supported by evidence from a systematic review of the literature, the phrase used here will read “Evidence‐ and consensus‐based (see Evidence Report)” instead of “Expert consensus”.

BOX 1 **The format for individual guideline recommendations, including strength of consensus and evidence.**

**Table:** 

**Should we … in chronic urticaria?**		
We **recommend** that …	↑↑	Strong consensus^1^ Expert consensus
^1^> 95% agreement

BOX 2 **The format for multiple guideline recommendations voted upon separately, including strength of consensus and evidence for each.**

**Table:** 

**Should we … in chronic urticaria?**		
We **recommend** that …	↑↑	Strong consensus^1^ Expert consensus
^1^> 95% agreement		
We **suggest** that …	↑	Strong consensus^1^ Expert consensus
^1^> 95% agreement

BOX 3 **The Format for Multiple Guideline Recommendations Voted on Jointly, Including Strength of Consensus and Evidence.**

**Table:** 

**Should we … in chronic urticaria?**		
We **recommend** that … We **recommend** using …	↑↑	Strong consensus^1^ Expert consensus
^1^> 95% agreement

In the event of multiple recommendations addressing the same question of interest and each of these recommendations having been voted upon separately, these can be grouped together as shown in Box 2. In this case, the strength of consensus and the evidence are given for each recommendation individually.

Box 3 shows an example when two recommendations are given instead of one. However, in this case, because these were voted on jointly in the consensus conference, the information on the strength of consensus and the evidence is shown only once and applies to both recommendations.

## 3 Definition

### 3.1 Definition

Urticaria is a condition characterized by the development of wheals (hives), angioedema, or both. Urticaria needs to be differentiated from other medical conditions where wheals, angioedema, or both can occur as features of a spectrum of clinical conditions such as anaphylaxis, autoinflammatory syndromes, urticarial vasculitis, or bradykinin‐mediated angioedema including hereditary angioedema (HAE).

Definition

Urticaria is a condition characterized by the development of wheals (hives), angioedema or both.

- A wheal has three typical features:
  1. a sharply circumscribed superficial central swelling of variable size and shape, almost invariably surrounded by reflex erythema,
  2. an itching or sometimes burning sensation,
  3. a fleeting nature, with the skin returning to its normal appearance, usually within 30 min to 24 h.
- Angioedema is characterized by:
  1. a sudden, pronounced erythematous or skin colored deep swelling in the lower dermis and subcutis or mucous membranes,
  2. tingling, burning, tightness, and sometimes pain rather than itch,
  3. a resolution slower than that of wheals (can take up to 72 h).

### 3.2 Classification of Urticaria Based on Its Duration and the Relevance of Eliciting Factors

The spectrum of clinical manifestations of different urticaria types and subtypes is extensive. Additionally, two or more different subtypes of urticaria can co‐exist in any patient. Urticaria is classified based on two main criteria: (i) its duration, as acute or chronic, and (ii) the role of definite triggers, categorized as inducible or spontaneous. **Acute urticaria** is defined as the occurrence of wheals, angioedema or both for 6 weeks or less. **Chronic urticaria (CU)** is defined as the occurrence of wheals, angioedema, or both for more than 6 weeks. CU can come with daily, or almost daily, signs and symptoms, or an intermittent/recurrent course. When the clinical course has no definite physical eliciting factor involved and shows a spontaneous course, it is described as **chronic spontaneous urticaria (CSU**). CSU episodes may recur after months or years of full remission [8].

Inducible urticaria is characterized by the presence of definite and subtype‐specific triggers leading to the development of wheals, angioedema, or both [9]. These triggers are definite because the symptoms always appear when the trigger at the individual threshold level is present and never when the trigger is absent. These triggers are specific because each subtype of inducible urticaria has its own relevant trigger, such as exposure to cold in cold urticaria, and this trigger is not relevant in other forms of inducible urticaria. Rare subtypes of inducible urticaria exist in which the combined presence of two or more specific triggers is required for the induction of the symptoms, such as in cold‐induced cholinergic urticaria [10]. In most types of **chronic inducible urticaria (CIndU)**, the symptoms appear usually within 10 min after exposure to the trigger and resolve within 1–3 h after cessation of exposure [11]. Rare subtypes of all inducible urticarias exist and cannot be discussed in detail in this guideline. Some patients suffering from CSU experience trigger‐induced augmentation of wheals, angioedema, or both. Among others, these triggers include stress and infections. Some patients can present with more than one subtype of urticaria, which can also respond independently to treatment [12].

**Table:** 

**How should urticaria be classified?**		
We **recommend** that urticaria is classified based on its duration as acute (≤ 6 weeks) or chronic (> 6 weeks)	↑↑	Strong consensus^1^ Expert consensus
^1^> 95% agreement		
We **recommend** that urticaria is classified as spontaneous (no definite eliciting factor involved) or inducible (specific definite factor involved)	↑↑	Strong consensus^1^ Expert consensus
^1^> 95% agreement		

Table 6 shows the classification of CU subtypes for clinical use. This classification has been maintained from the previous version of the guideline by strong consensus (≥ 95%).

**TABLE 6:** Recommended classification of chronic urticaria.

Chronic Urticaria Subtypes	
Chronic Spontaneous Urticaria (CSU)	Chronic Inducible Urticaria (CIndU)
Spontaneous appearance of wheals, angioedema or both for > 6 weeks due to known^a^ or unknown causes	Symptomatic dermographism^b^ Cold urticaria^c^ Delayed pressure urticaria^d^ Solar urticaria Heat urticaria^e^ Vibratory angioedema^f^ Cholinergic urticaria Contact urticaria Aquagenic urticaria

*Note:* Chronic urticaria (CU) is classified as spontaneous (CSU) and inducible (CIndU). CSU comes as CSU with known cause and CSU with unknown cause. CIndU is further subclassified as symptomatic dermographism, cold urticaria, delayed pressure urticaria, solar urticaria, heat urticaria, and vibratory angioedema (collectively referred to as chronic physical urticaria), as well as cholinergic urticaria, contact urticaria, and aquagenic urticaria. CU patients can concomitantly show more than one form of CU including more than one form of CIndU and they often do.

^a^ For example, Type I autoimmunity (autoallergy) and type IIb autoimmunity, with mast cell‐activating autoantibodies.

^b^ Formerly called *urticaria factitia* or dermographic urticaria.

^c^ Also called cold contact urticaria or acquired cold urticaria.

^d^ Also called pressure urticaria.

^e^ Also called heat contact urticaria.

^f^ Also called Vibratory angioedema/urticaria.

**Table:** 

**Should we maintain the current guideline classification of chronic urticaria?**		
We **recommend** that the current guideline classification of chronic urticaria should be maintained	↑↑	Strong consensus^1^ Expert consensus
^1^> 95% agreement		

Urticarial vasculitis, maculopapular cutaneous mastocytosis (formerly called urticaria pigmentosa), indolent systemic mastocytosis with involvement of the skin, mast cell activation syndrome (MCAS), auto‐inflammatory syndromes (e.g., cryopyrin‐associated periodic syndromes or Schnitzler's syndrome), nonmast cell mediator‐mediated angioedema (e.g., bradykinin‐mediated angioedema), and other diseases and syndromes that can manifest with urticaria‐like lesions and/or angioedema, are not considered to be types of urticaria due to their distinctly different pathophysiological mechanisms and/or clinical presentation (Table 7) [13, 14, 15, 16].

**TABLE 7:** Differential diagnoses of urticaria.

Maculopapular cutaneous mastocytosis (urticaria pigmentosa) and indolent systemic mastocytosis with involvement of the skin Mast cell activation syndrome (MCAS)
Urticarial vasculitis
Bradykinin‐mediated angioedema (e.g., HAE)
Exercise‐induced anaphylaxis
Cryopyrin‐associated periodic syndromes (CAPS; urticarial rash, recurrent fever attacks, arthralgia or arthritis, eye inflammation, fatigue and headaches), that is, Familial Cold Autoinflammatory Syndrome (FCAS), Muckle‐Wells Syndrome (MWS), or Neonatal Onset Multisystem Inflammatory Disease (NOMID)
Schnitzler's syndrome (recurrent urticarial rash and monoclonal gammopathy, recurrent fever attacks, bone and muscle pain, arthralgia, or arthritis and lymphadenopathy)
Gleich's syndrome (episodic angioedema with eosinophilia)
Well's syndrome (granulomatous dermatitis with eosinophilia/eosinophilic cellulitis)
Bullous pemphigoid (prebullous stage) Adult‐onset Still's disease (AOSD) Eosinophilic Dermatosis of Hematologic Malignancy (EDHM) Hypereosinophilic Syndromes (HES)
These differential diagnoses of urticaria are frequently misdiagnosed as urticaria because (1) they can present with wheals, angioedema, or both and/or (2) due to historical reasons. However, they are specific and distinct clinical entities

### 3.3 Pathophysiological Aspects

Urticaria is predominantly a mast cell‐driven disease but the triggers that activate mast cells are heterogeneous, diverse, and vary from patient to patient [17, 18]. Other than the histamine receptor, there are other receptors on mast cells that are also involved, such as the Mas‐related G‐protein coupled receptor member X2 (MRGPRX2) [19, 20, 21]. In addition to mast cells, several other cells and mediators can contribute to the pathology of CSU. These include T cells (i.e., interaction via OX40/OX40L, T cell cytokines), B cells (autoantibody production), eosinophils (i.e., release of MRGPRX2 agonists), neutrophils or basophils, macrophages, sensory nerves (i.e., release of neuropeptides), and others [9, 22]. In more than 50% of CSU patients, the pathophysiology is driven by two main autoimmune mechanisms: Type I (autoallergy) involving IgE autoantibodies against autoallergens, and type IIb involving IgG autoantibodies; a subpopulation of patients with CSU has both types [23, 24, 25]. Also, a significant number of CSU patients show signs of type 2 inflammation‐driven comorbidities [26]. However, it remains unclear how many of the factors and their interaction among each other are actually involved in mast cell activation in urticaria.‐ Overall, the result of mast cell activation is the release of mediators such as histamine and platelet activation factor (PAF), among others, which are responsible for sensory nerve stimuli (pruritus), vasodilatation (erythema), and inflammatory cells chemoattraction.

Histologically, wheals are characterized by degranulated mast cells, with edema of the upper‐ and mid‐dermis, with dilatation and augmented permeability of the postcapillary venules as well as lymphatic vessels of the upper dermis. In angioedema, similar changes occur primarily in the lower dermis and the subcutis. The skin affected by wheals shows a mixed inflammatory perivascular infiltrate of variable intensity, consisting of T cells, eosinophils, neutrophils, basophils, macrophages, and other cells. In urticarial wheals, a distinct feature of urticarial vasculitis characterized by vessel‐wall necrosis is not seen [13, 14]. However, in some cases of CSU with long‐standing wheals, a slight overlap may be observed on histopathology [27]. The non‐lesional skin of CSU patients has been shown to exhibit an upregulation of adhesion molecules, infiltrating eosinophils, altered cytokine expression [28, 29], and sometimes a mild to moderate increase of mast cell numbers [17]. These findings underline the complex nature of urticaria pathogenesis, which has many features in addition to the release of histamine from dermal mast cells [30, 31, 32]. Some of these features of urticaria are also observed in a wide variety of inflammatory conditions and are thus not specific or of diagnostic value. A search for more specific histological biomarkers for different subtypes of urticaria, and for distinguishing urticaria from other conditions, is necessary [33]. There is emerging evidence that changes in the gut microbiome may play a role in the pathophysiology of urticaria [34, 35]. However, as with the chicken or the egg dilemma, it is not clear if these changes are the cause or the consequence of the disease. However, insights from airway allergic diseases suggest that microbiome‐modulating interventions can have beneficial effects on the disease, as seen in rhinoconjunctivitis treatment [36]. Exploration of the microbiome's role in urticaria and the use of probiotics could open new avenues for disease management and treatment strategies [37].

### 3.4 Burden of Disease

Urticaria is a frequent disease affecting up to 20% of the world population at some point during their lifetime, with over half of those patients reporting angioedema, and the global prevalence continues to increase [37, 38]. The burden of CU on patients, their family and friends, the health care system, and society is substantial [38]. Its debilitating symptoms—severe pruritus and wheals—disruption of daily activities, sleep, and emotional well‐being can create a cycle of frustration and decreased quality of life (QoL) often worsened by comorbidities such as anxiety and depression [39].

Available data indicates that urticaria significantly impacts both objective functioning and subjective well‐being [40, 41, 42]. Health status scores in CSU patients have been shown to be comparable to those reported by patients with rheumatoid arthritis or insulin‐treated diabetes [43]. Furthermore, both health status and subjective satisfaction in CSU patients are lower than in healthy individuals and in patients with respiratory allergies [44]. CU also imposes considerable costs on patients and society [45, 46, 47].

## 4 Diagnosis of Urticaria

A detailed medical history is essential in the diagnosis of urticaria [48, 49] and is the first step in the diagnostic workup of all urticaria patients. This clinical anamnesis should include concomitant and comorbid diseases. The second step is the physical examination of the patient. Since wheals and angioedema are transient and may not be present at the time of physical examination, it is important to review prior documentation, including patient‐provided photographs of wheals and/or angioedema [50]. In CU, the third step involves a basic diagnostic workup which includes a limited set of recommended diagnostic tests (see Table 8). Additional and individually selected diagnostic tests may be useful based on the outcome of the first three steps and depending on the urticaria type and subtype (Table 8; extended diagnostic program).

**TABLE 8:** Recommended diagnostic tests in frequent urticaria subtypes.

Types	Subtypes	Routine diagnostic tests (recommended for every patient)	Extended diagnostic program^a^ (based on history)—For identification of underlying causes or eliciting factors and for ruling out possible differential diagnoses if indicated
Spontaneous urticaria	Acute spontaneous urticaria	None	None^b^
	CSU	Differential blood count. ESR and/or CRP IgG anti‐TPO and total IgE^c^	Avoidance of suspected triggers (e.g., drugs); Diagnostic tests for (in no preferred order): (i) infectious diseases (e.g., *Helicobacter pylori* ); (ii) functional autoantibodies (e.g., Autologous Serum Skin Test (ASST) and Autologous Plasma Skin Test (APST), basophil activation test); (iii) thyroid gland disorders (thyroid hormones and autoantibodies); (iv) allergy (skin tests and/or allergen avoidance test, e.g., avoidance diet); (v) concomitant CIndU^d^ (vi) severe systemic diseases (e.g., tryptase); (vii) other (e.g., lesional skin biopsy or lab test to exclude hereditary angioedema); (viii) C4 (screening) and C1INH (antigen and function) testing in patients with angioedema only phenotype
Inducible urticaria	Cold urticaria	Cold provocation and threshold test^d^, ^e^	Differential blood count and ESR or CRP, rule out other diseases, especially infections [51]
	Delayed pressure urticaria	Pressure test and threshold test^d^, ^e^	None
	Heat urticaria	Heat provocation and threshold test^d^, ^e^	None
	Solar urticaria	Provocation with UV and visible light of different wave lengths and threshold test^e^	Rule out other light‐induced dermatoses
	Symptomatic dermographism^f^	Elicit dermographism and threshold test^d^, ^e^	Differential blood count, ESR or CRP
	Vibratory angioedema	Test with vibration e.g., Vortex‐mixer^d^	None
	Aquagenic urticaria	Provocation testing^d^	None
	Cholinergic urticaria	Provocation and threshold testing^d^	None
	Contact urticaria	Provocation testing^d^	None

Abbreviations: CRP, C‐reactive protein; ESR, erythrocyte sedimentation rate.

^a^ Depending on suspected cause.

^b^ Unless strongly suggested by patient history, for example, allergy.

^c^ For patients in specialist care.

^d^ For details on provocation and threshold testing see [52].

^e^ All tests are done with different levels of the potential trigger to determine the threshold.

^f^ Symptomatic dermographism is characterized by wheals after shearing forces and has originally called dermographic urticaria.

It is imperative that the physician and patient have a clear understanding of the objectives of all diagnostic tests performed. It is of particular importance to find the balance between the necessary tests and the patient's wish to look for causative factors. It is also important to explain to the patient that CU is predominantly an autoimmune condition and that cases with positive results for IgE‐mediated food allergy do not necessarily indicate a causal relationship with urticaria, but may instead indicate a relevant comorbidity.

### 4.1 Diagnostic Workup in Acute Urticaria

Acute urticaria, because of its self‐limiting nature, usually does not require a diagnostic workup beyond a thorough medical history aimed at identifying possible trigger factors, such as recent infections at the time of onset (e.g., a common cold). The only exception to this is the suspicion of an acute urticaria due to a food allergy in sensitized patients, or drug hypersensitivity, especially for nonsteroidal anti‐inflammatory drugs (NSAIDs). In such cases, allergy tests, including provocation, can help to confirm a causative relation between the intake of such triggers and urticaria, as well as to find safe alternatives or tolerable doses in patients that use chronic NSAIDs due to other chronic medical conditions (i.e., coronary artery disease, rheumatoid arthritis). Patient education may also be useful to allow patients to avoid re‐exposure to the relevant causative factors. It is very important to differentiate acute urticaria from other conditions accompanied by wheals as a symptom, such as in acute anaphylaxis, where the symptoms do not persist for several days, unlike in acute urticaria. As with CU, other differential diagnoses need to be considered based on the patient's history.

**Table:** 

**Should routine diagnostic measures be performed in acute urticaria?**		
We **recommend against** routine diagnostic measures in acute spontaneous urticaria.	↓↓	Consensus^1^ Expert consensus
^1^> 75% agreement		

### 4.2 Diagnostic Workup in CSU

In CSU, the diagnostic workup has seven major aims: (i) to confirm the diagnosis and exclude differential diagnoses, especially for angioedema‐only patients; (ii) to look for the underlying causes; (iii) to identify relevant conditions that modify disease activity; (iv) to check for comorbidities; (v) to identify the consequences of CSU; (vi) to assess predictors of the course of disease and response to treatment; and (vii) to monitor disease activity, impact, and control (Table 9) [49].

**TABLE 9:** Aims of the diagnostic work up in patients with CSU [49].

What to do in every CSU patient			
History	Physical examination^a^	Basic tests^b^	UCT
**C**onfirm	Rule out differential diagnoses		
**C**ause	Look for indicators of CSU^aiTI^, CSU^aiTIIb^		
**C**ofactors	Identify potential disease triggers, aggravators, and modifying factors		
**C**omorbidities	For example, check for CIndU, related autoimmune conditions, mental health		
**C**onsequences	For example, evaluate sleep, mental health, sexual dysfunction, work, social performance		
**C**omponents	Assess potential biomarkers or predictors of treatment response		
**C**ourse	Monitor CSU activity, impact and control		

Abbreviations: CSU, chronic spontaneous urticaria; CSU^aiTI^, Type I autoimmune (autoallergic) CSU; CSU^aiTIIb^, Type IIb autoimmune CSU; UCT, urticaria control test.

^a^ Including review of patient photo documentation.

^b^ Differential blood count, CRP/Erythrocyte sedimentation rate; IgG‐anti‐TPO, total IgE for patients in specialist care.

In all CSU patients, the diagnostic workup should include a detailed medical history, physical examination (including review of pictures of wheals and/or angioedema), basic screening tests, and assessment of disease activity, impact, and control. To achieve this, the use of validated specific Patient Report Outcome Measures (PROMs) such as the Urticaria Activity Score over 7 days (UAS7), the Urticaria Control Test (UCT), and others, is very helpful. The basic tests include a differential blood count, CRP and/or ESR (to rule out severe systemic disease, as an indicator for parasitic disease, eosinophilic count, autoimmune disease such as systemic lupus), in all patients. In patients under specialist care, additional tests, such as total IgE and IgG‐anti‐TPO may be performed, as they indicate the likelihood of response to omalizumab [53]. Despite the established statistical association, the diagnostic performance and predictive accuracy of these tests remains limited [54]. Validated biomarkers assays are currently under development and are needed to better assist in decision‐making. Based on the results obtained by these measures, further diagnostic testing may be performed as outlined in Table 8.

#### 4.2.1 Confirmation of CSU and Exclusion of Differential Diagnoses

**FIGURE 1:**
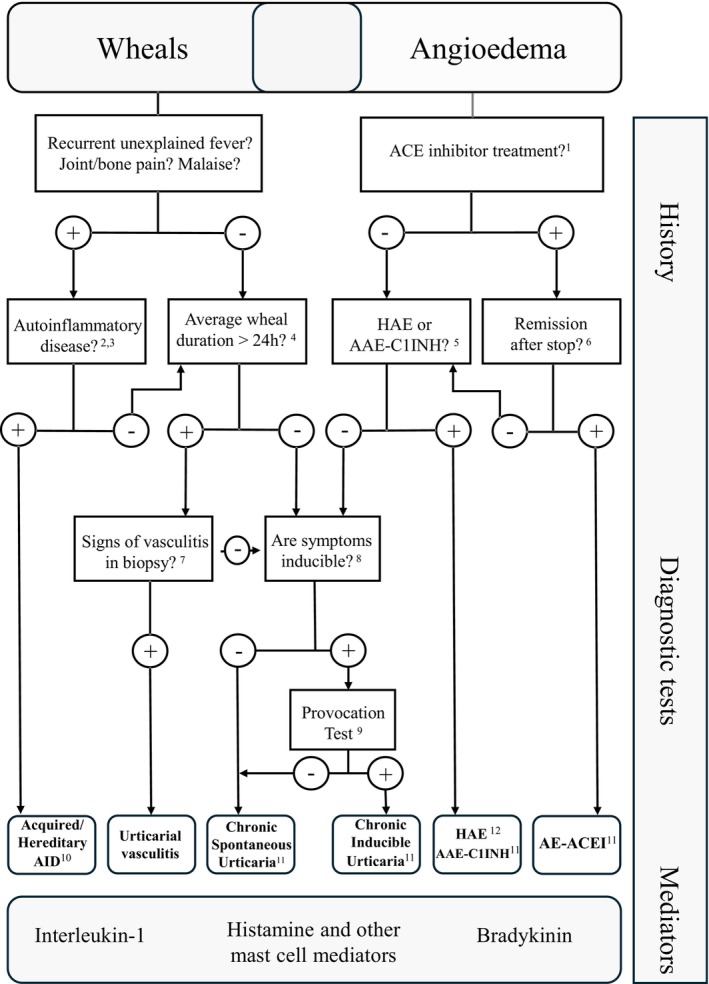
Diagnostic algorithm for patients presenting with wheals and/or angioedema. ^1^Apart from ACE inhibitors, angiotensin II type 1 receptor blockers (sartans), dipeptidyl peptidase IV inhibitors (gliptins) and neprilysin inhibitors have been described to induce angioedema when combined with ACE inhibitor but much less frequently. ^2^Patients should be asked for a detailed family history and age of disease onset. ^3^Test for elevated inflammation markers (C‐reactive protein, erythrocyte sedimentation rate), test for paraproteinemia in adults, look for signs of neutrophil‐rich infiltrates in skin biopsy; perform gene mutation analysis for hereditary periodic fever syndromes (e.g., Cryopyrin‐associated periodic syndrome), if strongly suspected. ^4^Patients should be asked: “For how long does each individual wheal last?” ^5^Test for Complement C4, C1‐INH levels and function; additionally test for C1q and C1‐INH antibodies, if AAE is suspected; do gene mutation analysis, if former tests are unremarkable but patient's history suggests hereditary angioedema. ^6^Remission should occur within a few days, in rare cases up to 6 months of ACE‐inhibitor discontinuation. ^7^Does the biopsy of lesional skin show damage of the small vessels in the papillary and reticular dermis and/or fibrinoid deposits in perivascular and interstitial locations suggestive of urticarial vasculitis? ^8^Patients should be asked: “Can you make your wheals appear? Can you bring out your wheals?” ^9^In patients with a history suggestive of inducible urticaria standardized provocation testing according to international consensus recommendations should be performed [40]. ^10^Acquired autoinflammatory syndromes include Schnitzler's syndrome as well as systemic‐onset juvenile idiopathic arthritis (sJIA) and adult‐onset Still's disease (AOSD); hereditary autoinflammatory syndromes include Cryopyrin‐associated periodic syndromes (CAPS) such as familial cold auto‐inflammatory syndromes (FCAS), Muckle‐Wells syndrome (MWS) and neonatal onset multisystem inflammatory disease (NOMID), more rarely hyper‐IgD syndrome (HIDS) and tumor necrosis factor receptor alpha‐associated periodic syndrome (TRAPS). ^11^In some rare cases recurrent angioedema is neither mast cell mediator‐mediated nor bradykinin‐mediated, and the underlying pathomechanisms remain unknown. These rare cases are referred to as “idiopathic angioedema” or “AE‐UNK” by some authors. ^12^Several subtypes HAE are known: HAE‐1: Hereditary angioedema due to C1‐Inhibitor deficiency; HAE‐2: Hereditary angioedema due to C1‐Inhibitor dysfunction; HAE nC1‐INH: Hereditary angioedema with normal C1‐Inhibitor levels, either due to a mutation in FXII (factor 12), ANGPT1 (angiopoietin‐1), PLG (plasminogen), KNG1 (kininogen), MYOF (myoferlin), HS3ST6 (heparan sulfate‐glucosamine 3‐O‐sulfotransferase 6), and CPN (carboxypeptidase N), or unknown. AAE‐C1INH, acquired angioedema due to C1‐inhibitor deficiency; AE, angioedema; AE‐ACEI, angiotensin converting enzyme inhibitor; AID, auto‐inflammatory disease; HAE, hereditary angioedema.

Wheals or angioedema also occur in patients with diseases other than CSU (Figure 1). In patients who exclusively develop wheals (but not angioedema), urticarial vasculitis [14], and autoinflammatory disorders [55], such as Schnitzler syndrome or cryopyrin‐associated periodic syndromes (CAPS), need to be ruled out. On the other hand, in patients who suffer exclusively from recurrent angioedema (but not from wheals), differential diagnosis should include bradykinin‐mediated angioedema, including those induced by angiotensin‐converting enzyme (ACE)‐inhibitor or gliptins, or hereditary angioedema (HAE) (Figure 1). The differential diagnoses of CSU is primarily guided by the patient's medical history (Figure 1) and is supported by tests, see Table 8.

**Table:** 

**Should differential diagnoses be considered in patients with chronic spontaneous urticaria?**		
We **recommend** that differential diagnoses be considered in all patients with signs or symptoms suggestive of chronic urticaria based on the guideline algorithm.	↑↑	Strong consensus^1^ Expert consensus
^1^100% agreement		
**What routine diagnostic measures should be performed in chronic spontaneous urticaria?**		
We **recommend** limited investigations. Basic tests include differential blood count, CRP and/or ESR, and in specialized care total IgE and IgG anti‐TPO, and more biomarkers as appropriate. We **recommend** performing further diagnostic measures based on the patient history and examination, especially in patients with long standing and/or uncontrolled disease.	↑↑	Consensus^1^ Expert consensus
^1^> 70% agreement		
**Should routine diagnostic measures be performed in inducible urticaria?**		
We **recommend** using provocation testing to diagnose chronic inducible urticaria. We **recommend** using provocation threshold measurements and the UCT to measure disease activity and control in patients with chronic inducible urticaria, respectively.	↑↑	Strong consensus^1^ Expert consensus
^1^> 95% agreement		

#### 4.2.2 Identification of Underlying Causes

Although CSU pathogenesis is not yet fully understood, it is well established that its signs and symptoms are due to the activation of skin mast cells and the subsequent release and effects of their mediators. Acute urticaria frequently follows upper airways infections [56], including COVID‐19 [57], and some of these cases evolve into CSU [58, 59]. In rare instances, chronic (silent) infections may contribute to CSU, and very rarely, an underlying malignancy has been reported [60]. However, it is not known how these infections relate to the recently identified causes of CSU, namely autoimmunity Type I (CSU^aiTI^, or “autoallergic CSU”; with IgE autoantibodies to self‐antigens) and autoimmunity Type IIb (CSU^aiTIIb^; with mast cell‐directed activating IgG autoantibodies). In CSU due to an unknown cause (CSU^uc^), unidentified mechanisms likely contribute to mast cell degranulation. The patient's history and physical examination can provide clues on the underlying causes. Basic tests findings can provide further guidance, for example, CSU^aiTIIb^ is often associated with elevated CRP and reduced eosinophil and basophil counts [61, 62]. Testing for IgG‐anti‐TPO and total IgE may also be more informative as CSU^aiTIIb^ patients are more likely to have low or very low total IgE and elevated levels of IgG‐anti‐TPO IgG [53].

However, although a clinical association between these laboratory markers and urticaria endotypes has been demonstrated, their clinical usefulness remains limited because there are no studies evaluating their diagnostic accuracy. Currently, cutoff points can be suggested but cannot be recommended for definitive endotype classification [54]. More advanced tests, such as basophil activation testing for CSU^aiTIIb^ can offer additional insights but are not routinely performed and should be guided by the patient's history, physical examination, and results of basic testing.

Other potential underlying causes include active thyroid disease, infections, cancer, inflammatory processes, food, and drugs, though these factors can be both causative as well as aggravating [48]. Intensive and costly general screening programs for causes of urticaria are not advised. Importantly, there may be considerable variations in the frequency of underlying causes in different parts of the world, and regional differences remain poorly researched and understood.

#### 4.2.3 Identification of Relevant Conditions That Aggravate Disease Activity

Identifying conditions and factors that aggravate CSU activity, such as certain medications, food, stress, and infections, can help both physicians and patients to understand and potentially change the course of CSU.

Medications can lead to urticarial drug reactions with wheals occurring directly after intake, based on both, the IgE‐mediated reaction type (e.g., penicillin), or non‐IgE‐mediated mechanisms (e.g., NSAID). Non‐IgE‐mediated reactions to drugs can also augment the activity of preexisting CSU. NSAIDs are the most common drugs to do so in up to one‐fourth of patients, except for paracetamol (acetaminophen) and/or COX‐2 inhibitors which are considered to be safer options in patients with CSU. Physicians should therefore ask patients about the intake of NSAIDs, including on‐demand use, and advise patients with a history to NSAID‐induced urticaria to avoid these drugs (NSAID challenges may be considered to avoid unnecessary restrictions when clinical history is not clear). However, if low‐dose acetylsalicylic acid is needed as an antithrombotic treatment, it is not always necessary to abstain from using this drug. A four‐week transition to another antithrombotic agent can help to assess causality, and if symptoms persist, reintroduction can be considered. Routine NSAID challenge testing is not recommended when the clinical history clearly indicated a reaction. Some studies suggest that NSAID challenges may be useful to assess tolerance once the disease is well controlled or in remission, however further studies are needed before a recommendation can be made in this regard [63].

Food allergy is rarely a trigger of CSU but needs to be ruled out as differential diagnoses in repeated episodes of short‐term wheals. However, food allergies may also coexist with urticaria as a comorbidity, such as in oral allergy syndrome (OAS). Furthermore, nonallergic reactions to food can trigger CSU exacerbation, and physicians should ask patients about such patterns. Based on this information, diagnostic diets such as pseudoallergen‐ and histamine‐low diets may be considered in selected patients as individualized diagnostic tools. Diagnostic diets should be maintained only for a limited time to avoid side effects and safety risks; 2–3 weeks are usually recommended. Importantly, diagnostic diets should not delay effective treatment. Importantly, the overdiagnosis of “histamine intolerance” should be avoided, as can lead to unnecessary dietary restrictions and misuse of commercial supplements.

It is evident that stress can exacerbate CSU, with up to one‐third of CSU patients perceiving stress as an aggravating factor of their disease [64, 65]. Physicians should ask patients about the impact of stress on their disease and make them aware that stress‐reducing interventions can be helpful.

#### 4.2.4 Identification of Comorbidities and Consequences of CSU

In CSU, the most common comorbidities are CIndUs, autoimmune diseases, metabolic syndrome, and allergies. Mental disorders, such as depression and anxiety, sexual dysfunction and sleep disturbance are also common consequences of CSU [66, 67]. Recent studies suggested an association between CSU and increased mortality, especially due to suicide, likely linked to its comorbidities [68]. Therefore, any findings from the patient's medical history, physical examination, or basic testing that suggest the presence of a comorbidity or consequence of CSU should prompt further investigations. These may include screening for specific diseases by questionnaires, provocation tests, further laboratory tests, or a referral to a specialist.

Several biomarkers have been identified as indicators of comorbidities, including other autoimmune conditions [69]. Table 10 lists several drugs used in the treatment of urticaria which have an effect on a comorbidity.

**TABLE 10:** Drugs used in the treatment of urticaria which have an effect on a comorbidity.

Comorbidity	Drug used in urticaria which has an effect on comorbidity
Asthma	Omalizumab, Dupilumab
Allergic rhinitis	H_1_‐antihistamine, Omalizumab
Atopic dermatitis	Dupilumab
Sinusitis/polyposis nasi	Omalizumab, Dupilumab
Food allergy	Omalizumab

#### 4.2.5 Identification of Predictors of the Course of Disease and Response to Treatment

In CSU, disease duration, disease activity, and response to treatment are linked to clinical characteristics and laboratory markers. While none of these are definite predictors, they can help physicians in counseling patients regarding disease severity, likely duration, and expected treatment outcomes.

For example, the presence of concomitant CIndU, high disease activity, elevated CRP and/or the presence of angioedema are indicative for a prolonged duration of CSU and suboptimal response to antihistamine treatment [70, 71, 72, 73].

#### 4.2.6 Assessment of Disease Activity, Impact, and Control

Patients should be assessed for disease activity, impact, and control at all visits. Validated PROMs, such as the urticaria activity score (UAS, and the weekly urticaria activity score, that is, UAS7, calculated from it), the cholinergic urticaria activity score (CholUAS), the angioedema activity score (AAS), the chronic urticaria quality of life questionnaire (CU‐Q2oL), the angioedema quality of life questionnaire (AE‐QoL), the urticaria control test (UCT), and the angioedema control test (AECT), should be used for this purpose [74, 75]. These tools are available in a wide range of languages.

For daily practice, recording the UAS, either on a sheet of paper or in a digital application (i.e., the CRUSE control application [76]), for a period of 1 month, offers valuable insight into disease fluctuations. While the UAS7 and other tools such as the UCT provide a mean score, daily tracking of the UAS can help to discover aggravating factors, such as NSAID use.

In CSU patients with wheals, disease activity should be assessed using UAS7 in both clinical care and clinical trials (Table 11). The UAS7 is a unified and simple scoring system that was proposed in the last version of the guideline and has been validated [77, 78]. It captures the two key signs and symptoms of urticaria—wheals and pruritus—based on daily self‐assessments by the patient, making it a particularly valuable tool. Using UAS7 enables consistent comparison of results across different studies and centers. Given the fluctuating nature of urticaria, the most reliable way to assess overall disease activity is through daily symptom tracking over several days. The UAS7, calculated as the sum of daily scores over 7 consecutive days, should be routinely used to monitor disease activity and treatment response in patients with CSU. For CSU patients who develop angioedema, with or without wheals, the Angioedema Activity Score (AAS) should be used to assess disease activity (Table 11) [79]. CSU patients who experience wheals and angioedema should use the UAS7 and the AAS in combination.

**TABLE 11:** The urticaria activity score (UAS) and Angioedema Activity Score (AAS) for assessing disease activity in CSU.

Urticaria Activity Score (UAS)		
Score	Wheals	Pruritus
0	None	None
1	Mild (< 20 wheals/24 h)	Mild (present but not annoying or troublesome)
2	Moderate (20–50 wheals/24 h)	Moderate (troublesome but does not interfere with normal daily activity or sleep)
3	Intense (> 50 wheals/24 h or large confluent areas of wheals)	Intense (severe pruritus, which is sufficiently troublesome to interfere with normal daily activity or sleep)
**Angioedema Activity Score (AAS)**		
**Score**	**Dimension**	**Answer options**
—	Have you had a swelling episode in the last 24 h?	No, yes
0–3	At what time(s) of day was this swelling episode(s) present? (please select all applicable times)	Midnight–8 a.m., 8 a.m.–4 p.m., 4 p.m.–midnight
0–3	How severe is/was the physical discomfort caused by this swelling episode(s) (e.g., pain, burning, itching?)	No discomfort, slight discomfort, moderate discomfort, severe discomfort
0–3	Are/were you able to perform your daily activities during this swelling episode(s)?	No restriction, slight restriction, severe restriction, no activities possible
0–3	Do/did you feel your appearance is/was adversely affected by this swelling episode(s)?	No, slightly, moderately, severely
0–3	How would you rate the overall severity of this swelling episode?	Negligible, mild, moderate, severe

For the UAS7 the sum of the score (0–3 for wheals +0–3 for pruritis) for each day is summarized over 1 week (7 days) for a maximum of 42. For the AAS, scores are summed up to an AAS day sum score (0–15), 7 AAS day sum scores to an AAS week sum score (AAS7, 0–105), and 4 ASS week sum scores may be summed up to an AAS 4‐week sum score (AAS28, 0–420). Copyright for UAS: GA^2^LEN; copyright for AAS (UK version): MOXIE GmbH (www.moxie‐gmbh.de).

In addition to the assessment of disease activity, it is important to evaluate the impact of the disease on the quality of life, as well as to determine the level of disease control both in clinical practice and in trials.

The CU‐Q2oL should be used to determine QoL impairment in CSU patients with wheals. For CSU patients with angioedema, with or without wheals, the AE‐QoL should be used. In CSU patients with wheals and angioedema, the CU‐Q2oL and the AE‐QoL should be used.

**FIGURE 2:**
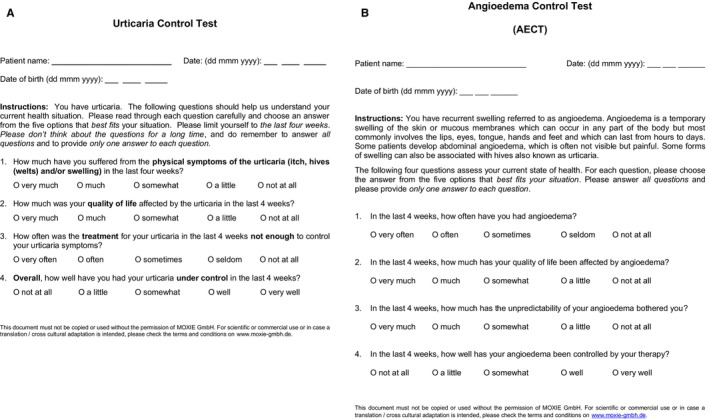
(A) The urticaria control test (UCT) and (B) The angioedema control test (AECT). Copyright: MOXIE GmbH, Berlin, Germany (www.moxie‐gmbh.de).

It is also important to assess disease control in patients with CSU. The Urticaria Control Test (UCT) should be used to do this in CSU patients who develop wheals, with or without angioedema (Figure 2A). For CSU patients who develop angioedema only, the Angioedema Control Test (AECT) should be used (Figure 2B). In CSU patients who develop wheals and angioedema, both the UCT and the AECT should be used.

The UCT was developed and validated to determine the level of disease control in all forms of CU (CSU and CIndU) [80, 81]. It is a simple four‐item tool with a clearly defined cut off for patients with “well‐controlled” vs. “poorly controlled” disease, and it is thus suited for the management of patients in routine clinical practice. Its recall period is 4 weeks. A 7‐day recall period UCT version is also available (UCT7) [82]. The UCT cutoff value for well‐controlled disease is 12 out of 16 possible points.

The AECT quantifies disease control in CSU patients with angioedema and patients with other forms of recurrent angioedema [74]. Like the UCT, the AECT is a retrospective PROM. Two versions of the AECT exist, one with a 4‐week recall period and one with a 3‐month recall period. The AECT consists, like the UCT, of only four questions. Its cut off for well controlled disease is 10 points. Both the UCT and the AECT are easy to administer and interpret. When completed, and the score is calculated, they can help to guide treatment decisions.

**Table:** 

**Should patients with chronic urticaria be assessed for disease activity, impact, and control?**		
We **recommend** that patients with CU be assessed for disease activity, impact, and control at every visit.	↑↑	Strong consensus^1^ Expert consensus
^1^100% agreement		
**Which instruments should be used to assess and monitor disease activity in chronic spontaneous urticaria patients?**		
We **recommend** the use of the urticaria activity score, UAS7, and/or of the angioedema activity score, AAS, for assessing disease activity in patients with chronic spontaneous urticaria.	↑↑	Strong consensus^1^ Expert consensus
^1^100% agreement		
**Which instruments should be used to assess and monitor quality of life impairment in chronic spontaneous urticaria patients?**		
We **recommend** the use of the chronic urticaria quality of life questionnaire, CU‐Q2oL, and the angioedema quality of life questionnaire, AE‐QoL, for assessing quality of life impairment in patients with chronic spontaneous urticaria.	↑↑	Strong consensus^1^ Expert consensus
^1^> 95% agreement		
**Which instruments should be used to assess and monitor disease control in chronic spontaneous urticaria patients?**		
We **recommend** the use of the urticaria control test, UCT, and/or the angioedema control test, AECT, for assessing disease control in patients with CSU.	↑↑	Strong consensus^1^ Expert consensus
^1^> 95% agreement		

### 4.3 The Diagnostic Workup in CIndU

In patients with CIndU, the routine diagnostic workup should adhere to the consensus recommendations regarding the definition, diagnostic testing, and management of CIndUs [52]. Diagnostic efforts in CIndU focus on three main objectives: to exclude differential diagnoses, to identify the subtype of CIndU, and to determine trigger thresholds [52]. The last of these is important as it allows for assessing disease activity and response to treatment.

For most CIndU subtypes, there are validated tools available for provocation testing [52]. Examples include cold and heat urticaria, for which a Peltier element‐based provocation device (Temp*Test*) is available [83], symptomatic dermographism for which dermographometers (Dermographic Tester, FricTest) [84, 85] have been developed, and delayed pressure urticaria tests (Dermographic Tester). In the case of cholinergic urticaria, a graded provocation test with office‐based methods, e.g., pulse‐controlled ergometry, is available [86, 87]. A new cholinergic urticaria activity score helps to identify everyday triggers [88]. Patients with contact urticaria [89] or aquagenic urticaria [90] should be assessed by appropriate cutaneous provocation tests.

Disease control in CIndU is assessed by provocation threshold testing and use of validated tools such as the UCT and AECT. PROMs for disease activity and impact are available, or being developed, for several CIndU subtypes [87, 91].

### 4.4 Diagnosis in Children

Urticaria can occur in individuals of all ages, including infants and young children. Recent reports indicate that, in children, the prevalence of CIndUs and CSU, as well as disease characteristics, underlying causes of CSU, and response to treatment, are very similar to those observed in adults [92, 93, 94, 95, 96, 97, 98, 99]. The diagnostic workup of CSU in children follows the same objectives as in adults. Differential diagnoses should be excluded with a special focus on cryopyrin‐associated periodic syndrome (CAPS), a rare autoinflammatory disease that presents with a urticaria‐like accompanied with fever that manifests in childhood [100].

If possible—depending on the age of the child, disease activity, impact and control should be assessed using assessment tools similar to those used in adults. While the UAS7 and UCT have been validated in children [101, 102], to date there are no other validated disease‐specific tools for children available. Triggers of exacerbation should be identified and, when indicated, investigating underlying causes—similar to those in adults—are important components of the diagnostic process. In children with CIndU, similar tests for provocation and the determination of trigger thresholds should be performed (as far as feasible given the child's age‐related ability to cooperate).

## 5 Management of Urticaria

### 5.1 Basic Considerations

**FIGURE 3:**
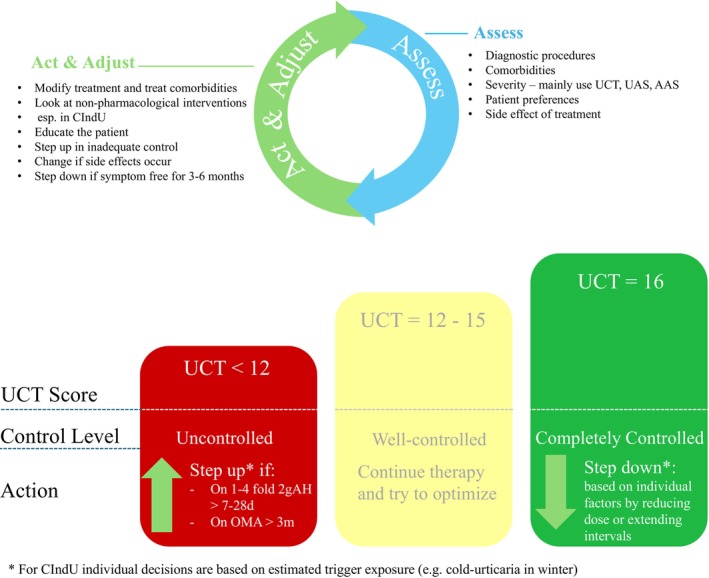
Chronic urticaria: management decisions and treatment adjustments. Apart from UCT, in some patients a monthly diary using the UAS and AAS can render better advice as this can discover short‐term exacerbations, especially although UCT states well controlled. In patients who are in the range of UCT 12–15, therefore a step up might also be required.

1. The goal of treatment is to treat the disease until it is gone, as efficiently and as safely as possible, aiming at a continuous complete control (consistently UAS = 0/UCT = 16) and a normalization of quality of life [103, 104].
2. The therapeutic approach to CU should involve
  - the search for, and if possible, elimination of underlying causes, which means healing the disease
  - the avoidance of eliciting factors, reducing disease activity
  - tolerance induction in some CIndUs, reducing disease activity
  - the use of pharmacological treatment to prevent mast cell mediator release and/or the effects of mast cell mediators, reducing disease activity
3. Treatment should follow the basic principles of treating as much as needed and as little as possible, taking into consideration that the activity of the disease may vary [105]. This implies stepping up or stepping down in the treatment algorithm according to the course of disease following the principle assess, act & adjust, and reassess (Figure 3). It is important to highlight that patients need good counseling regarding continuous treatment and using PROMs, especially about using the UAS.

**Table:** 

**Should treatment aim at complete symptom control in urticaria?**		
We **recommend** aiming at complete symptom control in urticaria, considering as much as possible the safety and the quality of life of each individual patient.	↑↑	Strong consensus^1^ Expert consensus
^1^> 95% agreement		

### 5.2 Identification and Elimination of Underlying Causes and Avoidance of Eliciting Factors

Although desirable, the elimination of underlying causes is currently not possible in most patients with urticaria. In the majority of patients, the underlying causes of CIndU and acute spontaneous urticaria remain unknown. Similarly, while several common underlying causes of CSU, such as Type I and Type IIb autoimmunity, have been identified, they are not curable at present. Although reduction of autoantibodies by plasmapheresis has demonstrated temporary benefit in some severely affected CSU patients [106], experience and evidence are limited and the costs of such a procedure are high.

In contrast, the avoidance of triggering factors, where possible, can be of benefit for patients with urticaria [107]. In CIndU, the avoidance of specific and definite triggers, such as cold in cold urticaria, can reduce disease activity. In CSU, the avoidance of individually relevant and unspecific triggers, for example stress or the intake of NSAIDs, can help to reduce disease exacerbations. It is crucial, however, to balance trigger avoidance with maintaining quality of life and participation in work and social activities, in patients with CIndU. For example, advising patients with cholinergic urticaria to completely avoid physical exercise or recommending that patients with solar urticaria avoid going outdoors should not be the aim of a patient‐centered management strategy.

#### 5.2.1 Drugs

When drugs are suspected as contributors during the diagnostic workup, they should be omitted entirely or substituted by another class of agents if indispensable. Drugs causing hypersensitivity reactions (especially NSAIDs) cannot only elicit but can also aggravate preexisting CSU. Consequently, elimination in the latter case will only partially improve symptoms in some patients.

**Table:** 

**Should patients with chronic spontaneous urticaria be advised to discontinue medication that is suspected to worsen the disease?**		
We **recommend** advising patients with chronic spontaneous urticaria to discontinue medication that is suspected to worsen the disease, e.g., NSAIDs.	↑↑	Strong consensus^1^ Expert consensus
^1^> 95% agreement		

#### 5.2.2 Definite and Specific Triggers of CIndU

Avoidance or reduction of the specific triggers of CIndUs can help to reduce the occurrence of wheals and angioedema. However, this approach is usually not sufficient to control the disease and can impose a substantial burden on patients. Patients should be provided with information that helps them to recognize and minimize relevant trigger exposure. For example, patients with delayed pressure urticaria should be informed that pressure is defined as force per area and that simple measures, such as broadening of the handle of heavy bags, may be helpful in the prevention of symptoms. Similar considerations hold for cold urticaria where the impact of the wind chill factor in cold winds needs to be remembered. For solar urticaria, the exact identification of the range of eliciting wave lengths may be important for the appropriate selection of sunscreens or light bulbs with a UV‐A filter. However, in many patients, the threshold for the relevant physical trigger is low and total avoidance of symptoms is virtually impossible. For example, severe symptomatic dermographism is sometimes confused with CSU because seemingly spontaneous hives are observed where even loose‐fitting clothing rubs on the patient's skin, or unintentional scratching by the patient readily causes the development of wheals in that area.

#### 5.2.3 Infections and Inflammatory Processes

In contrast to CIndU, CSU has been reported to be associated with a variety of inflammatory or infectious diseases, as well as vaccinations [58, 108]. This is regarded as significant in some instances, but studies show conflicting results and have methodological weaknesses. Infections that may contribute to CSU disease activity include those of the gastrointestinal tract such as an *H. pylori* infection, Lyme disease, and bacterial infections of the nasopharynx [109, 110, 111, 112, 113]. Even though the association with urticaria is not clear in the individual patient and a meta‐analysis shows overall low evidence for eradication therapy [109], *H. pylori* should be eliminated as an association with gastric cancer is suggested [114]. Furthermore it has been shown that the infection with *H. pylori* itself is not the trigger, but rather the resulting gastritis and urticaria will only subside if this is also sufficiently treated [110]. Bowel parasites, a rare possible cause of CSU in developed industrial countries, should be eliminated if indicated [109, 115, 116]. In the past, intestinal candidiasis was regarded as an important underlying cause of CSU [109], but more recent findings fail to support a significant causative role [117]. Apart from infectious diseases, chronic inflammatory processes due to other diseases have been identified to potentially trigger CSU. These can be secondary to infections. This holds true particularly for gastritis, reflux esophagitis, or the inflammation of the bile duct or gall bladder [118, 119]. Thus it could be shown that successful eradication of *H. pylori* is only having an impact on CSU if also the subsequent inflammation, that is, gastritis and esophagitis, is healed [110]. However, similar to infections, it is not easily possible to discern whether any of these are relevant causes of CSU, but should be treated regardless since many of them may be also associated with the development of malignancies.

#### 5.2.4 Stress

Although the mechanisms of stress‐induced exacerbation are not well investigated, some evidence indicates that disease activity in patients with CSU can be linked to stress [65, 120]. Further studies are needed to characterize the prevalence and relevance of CSU exacerbation by stress as well as the underlying mechanisms. In a patient‐centered approach the discussion of stress and stress‐avoidance is part of the management [64].

#### 5.2.5 Reduction of Functional Autoantibodies

Direct reduction of functional autoantibodies by plasmapheresis has been shown to be of temporary benefit in some, severely affected patients [106]. Due to limited experience and high costs, this therapy is suggested for autoantibody‐positive CSU patients who are unresponsive to all other forms of treatment. Autoantibodies and potentially activated T cells may also be reduced by immunosuppressive medication, such as cicloporin [121].

#### 5.2.6 Food

IgE‐mediated food allergy is extremely rarely the underlying cause of CSU [119, 122]. If identified, the specific food allergens need to be omitted as much as possible, which leads to a remission within less than 24 h. In some CSU patients, pseudoallergic reactions (non‐IgE‐mediated hypersensitivity reactions) to naturally occurring food ingredients, and in some cases to food additives, have been observed [119, 122, 123, 124, 125, 126]. A pseudoallergen‐low diet, containing only low levels of natural as well as artificial food pseudoallergens, has been tested in different countries [127]. Also, a low histamine diet may improve symptoms in some patients [128]. Those diets have been criticized and have not been proven in well‐designed double‐blinded placebo‐controlled studies, which however are not possible as the diet needs to be monitored for 14 days. However, all open label studies have shown to be beneficial. This kind of treatment has the advantage that it does not involve costs for medications which is relevant in places without reimbursement, but it also requires cooperative patients and success rates may vary considerably due to regional differences in food and dietary habits. More research is necessary on the effects of natural and artificial ingredients of food on urticaria.

### 5.3 Inducing Tolerance

Inducing tolerance can be useful in some subtypes of CIndU. Examples are cold urticaria, cholinergic urticaria, and solar urticaria, where a rush therapy with UV‐A has been reported to be effective within 3 days [129]. However, tolerance induction is only lasting for a few days, thus a consistent daily exposure to the stimulus just at threshold level is required. Tolerance induction and maintenance are often not accepted by patients, for example, in the case of cold urticaria where daily cold baths/showers are needed to achieve this.

### 5.4 Symptomatic Pharmacological Treatment

*The aim and the need for continued but adjusted treatment*

The overall aim of the management in urticaria is to achieve complete control of symptoms and maintain effective and safe treatment until the disease is gone. This includes nonpharmacological measures like avoidance of triggers, as described above, in combination with pharmacological treatment using the minimal effective dose of the drug, or combination of drugs, that help the patient to maintain complete control. In order to constantly ensure the right level of treatment, patients need to be seen at regular intervals and adjustments are recommended based on the level of symptoms. For updosing, also the time of onset needs to be considered, for example, for treatment with an antihistamine, further improvement cannot be expected after 1–2 weeks of treatment as receptor saturation is complete after 1 week. For a stepdown, different options exist but costs are especially relevant for choosing which step to take first.

*The targets*

Current recommended and effective treatment options for urticaria aim to target mast cell mediators at the receptors on nerves and endothelia, mast cell releasability, or mast cell activation by autoimmune triggers among others. Novel treatments currently under development aim to better regulate Type 2 immunity, or silence mast cells via inhibitory receptors, or to reduce mast cell numbers. The overall goal of all these symptomatic treatments is to help patients to be free of signs and symptoms until their episode of urticaria shows complete symptom control under treatment and in the future, ideally to create a curative pharmacological approach. To achieve this, pharmacological treatment should be continuous, until no longer needed. Nonsedating 2nd generation H_1_‐antihistamines, for example, should be used daily, to prevent the occurrence of wheals and angioedema, rather than on demand. This has been so far supported by their safety profile (safety data are available for several years of continuous use and in pregnancy), the results of randomized controlled trials and real‐life studies [130, 131, 132], and their mechanism of action, that is, their inverse agonist effects on the H_1_ receptor, stabilizing its inactive state. Although continuous use of active treatment is also recommended, especially in very active and uncontrolled CIndU, some patients can benefit from short term prophylactic antihistamine treatment before a relevant trigger exposure. However, in patients with CIndU, an individualized decision should be made, based on the presence and predictability of triggers, as to whether 2nd generation H_1_‐antihistamines are taken regularly or as needed.

*H*_*1*_ *‐antihistamine treatment*

H_1_‐antihistamines have been available for the treatment of urticaria since the 1950s. The older 1st generation H_1_‐antihistamines have pronounced anticholinergic and sedative effects and many interactions with alcohol and other drugs such as analgesics, hypnotics, sedatives, and mood elevating drugs, have been described. They can also interfere with rapid eye movement (REM) sleep and impact learning and performance. Impairment is particularly prominent during multi‐tasking and performance of complex sensorimotor tasks, such as driving. In a GA^2^LEN position paper [133] it is strongly recommended not to use 1st generation H_1_‐antihistamines any longer in allergy for adults and especially not for children. This view is shared by the WHO ARIA guideline [134]. Based on strong evidence regarding potentially serious side‐effects of 1st generation H_1_‐antihistamines (lethal overdoses have been reported) [135], we recommend against their use for the routine management of CU as first‐line agents [136].

Modern 2nd generation H_1_‐antihistamines are minimally or nonsedating and free of anticholinergic effects [137]. However, two 2nd generation H_1_‐antihistamines, astemizole, and terfenadine, are shown to have cardiotoxic effects in patients treated with inhibitors of the cytochrome P450 (CYP) 3A4 isoenzyme, such as ketoconazole or erythromycin. Astemizole and terfenadine are no longer available in most countries, and we recommend that they are not used.

Most, but not all 2nd generation H_1_‐antihistamines have been tested specifically in urticaria, and evidence supports the use of bilastine, cetirizine, desloratadine, ebastine, fexofenadine, levocetirizine, loratadine, mizolastine, and rupatadine [132, 138, 139]. We recommend the use of a standard‐dosed modern 2nd generation H_1_‐antihistamines as the first‐line symptomatic treatment for urticaria. However, no recommendation can be made on which to choose because, to date, well‐designed clinical trials directly comparing the efficacy and safety of all modern 2nd generation H_1_‐antihistamines in urticaria are largely lacking. Considering treatment adjustments, it is important to know that the onset of action is quick and that doses, especially in CIndU, can be adjusted according to need, for example, outside temperature in cold urticaria and there is no tachyphylaxis. Even though rarely, some patients suffer urticaria exacerbations from using H_1_‐antihistamines and these patients are candidates to be treated with omalizumab [140].

Further information as well as a systematic presentation of the processed evidence underlying the treatment recommendation can be found in the evidence report (pp. 5–42).

**Table:** 

**Should modern 2** ^**nd**^ **generation H**_**1**_ **‐antihistamines be used as first‐line treatment of urticaria?**		
We **recommend** a 2^nd^ generation H_1_‐antihistamine as first‐line treatment for all types of urticaria.	↑↑	Strong consensus^1^ Evidence‐ and consensus‐based (see Evidence Report, pp. 5–20)
^1^100% agreement		
**Is an increase in the dose to up to four‐fold of modern 2** ^**nd**^ **generation H**_**1**_ **‐antihistamines useful and to be preferred over other treatments in urticaria?**		
We **recommend** updosing of a 2^nd^ generation H_1_‐antihistamine up to 4‐fold in patients with chronic urticaria unresponsive to a standard‐dosed 2^nd^ generation H_1_‐antihistamines as second‐line treatment before other treatments are considered.	↑↑	Strong consensus^1^ Evidence‐ and consensus‐based (see Evidence Report, pp. 24‐27, 31‐39)
^1^> 95% agreement		
**Should modern 2** ^**nd**^ **generation H**_**1**_ **‐antihistamines be taken regularly or as needed?**		
We **suggest** 2^nd^ generation H_1_‐antihistamines to be taken regularly for the treatment of patients with chronic spontaneous urticaria. For the treatment of patients with chronic inducible urticaria, we **suggest** to decide based on the presence of triggers (e.g., time of the year in cold urticaria) whether 2nd generation H1‐antihistamines should be taken regularly or as needed.	↑	Strong consensus^1^ Evidence‐ and consensus‐based (see Evidence Report, pp. 21–23)
^1^> 95% agreement		
**Should different 2** ^**nd**^ **generation H**_**1**_ **‐antihistamines be used at the same time?**		
We **suggest against** using different H_1_‐antihistamines at the same time (against an updosing of a single H_1_‐antihistamine if not legally restricted in country)	↓	Consensus^1^ Evidence‐ and consensus‐based (see Evidence Report, pp. 28–30, 40–42)
^1^> 95% agreement		

Several studies show the benefit of the use of a higher than standard dose 2nd generation H_1_‐antihistamines in urticaria patients [141, 142, 143] corroborating earlier studies with 1st generation H_1_‐antihistamines which have concluded the same result [144, 145]. Studies support the use of up to fourfold standard‐dosed bilastine, cetirizine, desloratadine, ebastine, fexofenadine, levocetirizine, mizolastine, and rupatadine [132, 138, 139, 141, 142, 146, 147, 148, 149, 150].

Further information as well as a systematic presentation of the processed evidence underlying the treatment recommendation can be found in the evidence report (p. 49).

**Table:** 

**If there is no improvement, should higher than fourfold doses of 2** ^**nd**^ **generation H**_**1**_ **‐antihistamines be used?**		
We **recommend against** using higher than 4‐fold standard dosed H_1_‐antihistamines in chronic urticaria	↓↓	Strong consensus^1^ Evidence‐ and consensus‐based (see Evidence Report, p. 49)
^1^> 95% agreement		

**FIGURE 4:**
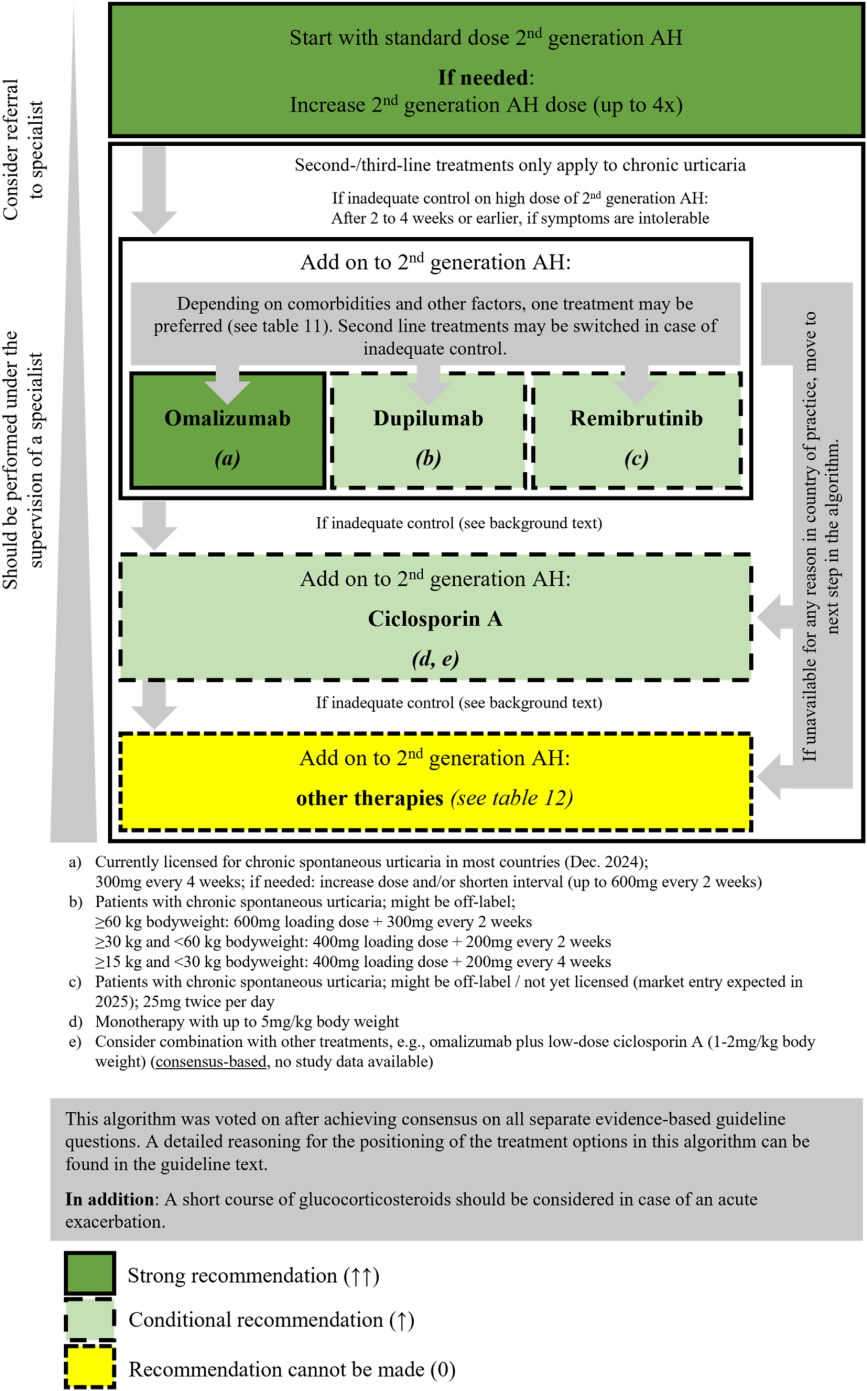
Recommended treatment algorithm for urticaria. This algorithm outlines the stepwise management approach for urticaria, developed in accordance with clinical evidence evaluated using the GRADE methodology. Each treatment recommendation in the management section (Section 5) was formally endorsed through expert voting, as indicated in the corresponding boxes. AH, antihistamine.

In summary, these studies suggest that some patients with urticaria, who show insufficient response to a licensed‐dose of 2nd generation H_1_‐antihistamine, benefit from updosing, which is preferred over mixing different 2nd generation H_1_‐antihistamines since their pharmacologic properties are different. The clinical advantage of updosing over multiple combinations of substances is also supported empirically in CU patients [150]. We, therefore, recommend to increase the dose up to fourfold, in such patients (Figure 4). Patients need to be informed that 2nd generation H_1_‐antihistamine updosing is off‐label and higher than fourfold is not recommended as it has not been tested. However, updosing has been suggested in the guidelines for urticaria since the year 2000, and so far, no serious adverse events have been reported, nor has a side effect ever been reported in the literature attributed to long‐term intake and potential accumulation. In a population‐based study in Taiwanese patients with new‐onset allergic rhinitis (2011–2017) using first and 2nd‐generation H_1−_antihistamines compared to non‐antihistamine users, showed that patients with allergic rhinitis on 1st‐ or 2nd‐generation H_1_‐antihistamines face an escalating dementia risk with increasing cumulative dosage [151]. It should be noted that the study's methodology is not sufficiently robust to challenge existing guideline recommendations. For example, the sample was heavily skewed towards patients taking antihistamines, which could have reduced the sensitivity in detecting any statistically significant increase in dementia risk among nonusers. In a later analysis of TriNetX data (presented at the GUF 2024 conference, data not published), the relationship was not confirmed. In addition, a separate study reported improved mental health outcomes associated with the use of first‐generation antihistamines [136].

In conclusion, we regard the results of this study as a safety signal which needs further monitoring but does not alter our current evaluation of the benefit and risk potential of the use of second‐generation antihistamines in CSU.

*Omalizumab treatment*

Nearly worldwide, omalizumab (monoclonal anti‐IgE antibody) is the only other licensed treatment in urticaria. It is for patients who do not show sufficient benefit from treatment with a 2nd generation H_1_‐antihistamine, and therefore it is recommended as the next step in the algorithm. Omalizumab has been shown to be very effective and safe in the treatment of CSU [152, 153, 154, 155, 156, 157, 158, 159, 160]. Omalizumab has also been reported to be effective in CIndU [161, 162, 163, 164] including cholinergic urticaria [165], cold urticaria [166, 167], solar urticaria [168], heat urticaria [169], symptomatic dermographism [170, 171] as well as delayed pressure urticaria [172]. In CSU, omalizumab prevents wheal and angioedema development [173, 174], markedly improves quality of life [175, 176], is suitable for long‐term treatment, and effectively treats relapse after discontinuation [177, 178, 179]. The recommended initial dose in CSU is 300 mg every 4 weeks as add on to antihistamine treatment. Dosing is independent of total serum IgE and bodyweight [152].

Patients with urticaria who do not show sufficient benefit from treatment with omalizumab at the licensed dose of 300 mg every 4 weeks can be treated with omalizumab at higher doses, shorter intervals, or both. Studies support the use of omalizumab treatment at doses up to 600 mg and intervals of 2 weeks, in patients with insufficient response to standard dosed omalizumab [180, 181, 182, 183]. Patients need to be informed that omalizumab updosing is off‐label. But also, the prolongation of the interval is strictly off label.

When considering treatment adjustments, it is important to note that the onset of action is often slow. Doses, especially in CIndU, can be adjusted according to need, for example, the time of the year in cold urticaria, or in the case of symptom absence in CSU, a prolongation of the interval should be considered. After 3 months of complete control, the interval can be prolonged until long as first wheals reappear, as there is no tachyphylaxis nor have relevant levels of blocking antibodies been described.

Drug survival studies, or time to discontinuation, for omalizumab show that the main reason for discontinuation is due to well‐controlled disease and far less due to ineffectiveness or adverse effects [178, 181].

Further information as well as a systematic presentation of the processed evidence underlying the treatment recommendation can be found in the evidence report (pp. 49–60).

**Table:** 

**Is omalizumab useful as add‐on treatment in patients unresponsive to high doses of H**_**1**_ **‐antihistamines?**		
We **recommend** adding on omalizumab* for the treatment of patients with CU unresponsive to high doses of 2^nd^ generation H_1_‐antihistamines. *Currently licensed for chronic spontaneous urticaria	↑↑	Strong consensus^1^ Evidence‐ and consensus‐based (see Evidence Report, pp. 49–60)
^1^> 95% agreement		

*Dupilumab treatment*

Several medications, already proven effective for other conditions, are currently studied for their potential benefits in treating antihistamine‐refractory CSU. This also pertains to the monoclonal anti‐IL4Rα antibody, dupilumab, that blocks the effect of IL‐4 and IL‐13. Results of Phase 3 studies show the efficacy of dupilumab as add‐on treatment, which is however, likely to be less pronounced than those observed with omalizumab [184]. Currently, urticaria‐specific long‐term safety data are lacking, but existent studies in various diseases reflect a generally favorable safety profile [185, 186, 187, 188, 189, 190, 191, 192, 193, 194]. Available data shows efficacy regardless of low baseline IgE levels for dupilumab, which is different compared to omalizumab where very low IgE values are often a reason for insufficient response [184]. Dupilumab can offer synergistic values to treat urticaria in conjunction with certain comorbidities such as asthma, chronic rhinosinusitis with nasal polyps, and atopic dermatitis. Dupilumab is approved for the treatment of urticaria in the USA, UAE, Brazil, and Japan where it is already licensed, and licensing in other countries is expected in 2025.

Further information as well as a systematic presentation of the processed evidence underlying the treatment recommendation can be found in the evidence report (pp. 61–63).

**Table:** 

**Is dupilumab useful as add‐on treatment in patients unresponsive to high doses of H**_**1**_ **‐antihistamines?**		
We **suggest** using dupilumab* as add‐on treatment for patients with CSU unresponsive to high doses of 2nd generation H1‐antihistamines. *might be off‐label	↑	Consensus^1^ Evidence‐ and consensus‐based (see Evidence Report, pp. 61–63)
^1^> 75% agreement		

*Remibrutinib treatment*

The clinical efficacy from Phases 2 and 3 studies of the Bruton's tyrosine kinase inhibitor, remibrutinib, as an add‐on treatment to H1‐antihistamines has recently been demonstrated for CSU and also suggests a favorable safety profile [195, 196, 197]. In September 2025 remibrutinib was approved by the FDA as an oral treatment for adult patients with CSU who remain symptomatic despite H1‐antihistamine treatment and does not require injections or lab monitoring [198]. Approval in other countries is expected soon. The use of remibrutinib can be considered in all patients with CSU and especially in patients who do not respond to both high‐dose antihistamine and omalizumab treatment. The available high‐quality data on its efficacy and the current and expected licensing, warrant the drug to be included in the treatment algorithm. Although other dosages have demonstrated benefits in a dose‐finding study, 25 mg twice daily will most likely be recommended as the treatment dose based on superior efficacy data [196].

Further information as well as a systematic presentation of the processed evidence underlying the treatment recommendation can be found in the evidence report (pp. 64–85).

**Table:** 

**Is remibrutinib useful as add‐on treatment in patients unresponsive to high doses of H**_**1**_ **‐antihistamines?**		
We **suggest** using remibrutinib* as add‐on treatment for patients with CSU unresponsive to high doses of 2nd generation H1‐antihistamines.	↑	Consensus^1^ Evidence‐ and consensus‐based (see Evidence Report, pp. 64–85)
^1^> 75% agreement		

*Ciclosporin treatment*

Patients with CSU who do not show sufficient benefit from treatment with H1‐antihistamines, especially if they are also unresponsive to omalizumab, can alternatively be treated with ciclosporin 3–5 mg/kg per day. Ciclosporin is immunosuppressive and has a moderate and direct effect on mast cell mediator release [199, 200]. Efficacy of ciclosporin in combination with a modern 2^nd^ generation H_1_‐antihistamine has been shown in placebo‐controlled trials [121, 201, 202] as well as open controlled trials in CSU [203], but this drug cannot be recommended as standard treatment due to a higher incidence of adverse effects, especially with long‐term use [201]. Ciclosporin is off‐label for urticaria and is recommended only for patients with severe disease refractory to any other licensed treatments. Ciclosporin can be especially effective in selected patients with CSUaiTIIb with low total IgE levels and other markers of autoimmunity [204]. Based on a small retrospective study with no control group, short courses of ciclosporin may induce long remissions in some CSU patients [205].

However, ciclosporin has a far better risk/benefit ratio compared to long‐term use of steroids. Some physicians also use tacrolimus which has a very similar mode of action with a better adverse event profile but there are no studies available which would allow for any recommendation here.

Further information as well as a systematic presentation of the processed evidence underlying the treatment recommendation can be found in the evidence report (pp. 94–96).

**Table:** 

**Is ciclosporin useful as add‐on treatment in patients unresponsive to high doses of H**_**1**_ **‐antihistamine?**		
We **suggest** using ciclosporin* for the treatment of patients with CU unresponsive to licensed treatments or if these are not available. *currently not licensed for CU	↑	Consensus^1^ Evidence‐ and consensus‐based (see Evidence Report, pp. 94–96)
^1^> 75% agreement		

*Other symptomatic treatments*

Some previous randomized clinical trials have assessed the use of leukotriene receptor antagonists. Studies are difficult to compare due to different populations studied, for example, inclusion of only aspirin and food additive intolerant patients or exclusion of patients with positive autologous serum skin test results. In general, the level of evidence for the efficacy of leukotriene receptor antagonists in urticaria is low, but the most so far for montelukast. A recent, systematic review and meta‐analysis found that adding leukotriene receptor antagonists to H1 antihistamines provides modest reductions in urticaria activity without significantly increasing overall adverse events, though the risk of neuropsychiatric effects remains uncertain [206]. Therefore, it is highly important to advert patients on the rare, but confirmed possible neuropsychiatric adverse events associated with montelukast [207], generally during the first days or weeks of treatment, due to which the drug has a black‐boxes warning by the FDA. At present, topical corticosteroids are frequently and successfully used in many allergic diseases, but are not useful in urticaria (with the possible exception of pressure urticaria on soles as alternative therapy with low evidence). If systemic corticosteroids are used, doses between 20–50 mg/day of prednisone equivalent are needed (dose is appropriate for adults and not children). Because such high doses will have side effects over the long term, we strongly recommend against the prolonged use of corticosteroids. Depending on the country it must be noted that steroids are also not licensed for CU (e.g., in Germany and Japan, prednisolone is only licensed for acute urticaria). For acute urticaria and acute exacerbations of CSU, a short course of oral corticosteroids, that is, treatment of a maximum of up to 10 days, may, however, be helpful to reduce disease duration/activity [208, 209]. Nevertheless, well‐designed randomized clinical trials are lacking.

Further information as well as a systematic presentation of the processed evidence underlying the treatment recommendation can be found in the evidence report (p. 103).

**Table:** 

**Should oral corticosteroids be used as add‐on treatment in the treatment of urticaria?**		
We **recommend against** the long‐term use of systemic glucocorticosteroids or depot preparations in CU.	↓↓	Strong consensus^1^ Evidence‐ and consensus‐based (see Evidence Report, p. 103)
We **suggest** considering a short course of rescue systemic glucocorticosteroids in patients with an acute exacerbation of CU.	↑	
^1^> 95% agreement		

While H1 antihistamines at up to four‐fold the manufacturers' recommended dosages may control symptoms in a large proportion of patients with urticaria in general practice, alternative treatments are needed for the remaining unresponsive patients. It is strongly recommended to stick to the treatment algorithm, but it is acknowledged that omalizumab, dupilumab, and remibrutinib have restrictions due to their high cost, and that ciclosporin restricted due to its safety profile. Furthermore, licensing issues and economic cost must be considered with any intervention and alternative treatment options should be considered.

Since the severity of urticaria may fluctuate, and spontaneous remission may occur at any time, it is also recommended to re‐evaluate the necessity for continued or alternative drug treatment every three to 6 months. This is also reflected in Figure 3.

Almost all treatments not listed in the treatment algorithm (Figure 4) are based on clinical trials providing low levels of evidence. As an exception, moderate quality evidence exists for the monoclonal antibody benralizumab, which failed to show statistically significant efficacy in a classical trial of CU but may be considered in specific patients especially if certain comorbidities, such as eosinophilic asthma are present (Table 12) [210, 211].

**TABLE 12:** Alternative treatment options.

*Although evidence from publications is low, clinical experience indicates that they may be useful in certain contexts. Interventions are listed in alphabetical order by frequency of use rather than efficacy*		
Intervention	Substance (class)	Possible indication
*Widely used*		
Tricyclic antidepressant with potent H1‐ and H2‐antagonist activity	Doxepin^a^	CSU, Idiopathic cold‐induced urticaria
Diet	Pseudoallergen‐low diet^b^	CSU
H_2_‐antihistamine	Ranitidine^c^, famotidine, Lafutidine (available in Japan, South Korea, and India)	CSU
Immunosuppressive	Methotrexate Mycophenolate mofetil	CSU ± DPU^d^ Autoimmune CSU
Leukotriene receptor antagonist	Montelukast	CSU, DPU
Sulphones	Dapsone, Sulphasalazine	CSU ± DPU CSU ± DPU
*Infrequently used*		
Anabolic steroid	Danazol	Cholinergic urticaria
Anticoagulant	Warfarin	CSU
Antifibrinolytic	Tranexamic acid	CSU with angioedema
Immunomodulator	IVIG Plasmapheresis	Autoimmune CSU Autoimmune CSU
Miscellaneous	Autologous blood/serum Hydroxychloroquine	CSU CSU
Phototherapy	Narrow‐band UVB	Symptomatic dermographism
Psychotherapy	Psychotherapy	CSU
*Rarely used*		
Anticoagulant	Heparin	CSU (elevated D‐Dimer)
Immunosuppressive	Cyclophosphamide Rituximab	Autoimmune CSU Autoimmune CSU
Miscellaneous	Anakinra Anti‐TNF‐alpha Camostat mesilate Colchicine Miltefosine Mirtazapine PUVA Benralizumab^e^	DPU CSU ± DPU CSU CSU CSU CSU CSU CSU ± eosinophilic asthma
*Very rarely used*		
Immunosuppressive	Systemic Tacrolimus	CSU
Miscellaneous	Vitamin D Interferon alpha	CSU CSU

Abbreviations: CSU, Chronic Spontaneous Urticaria; DPU, Delayed Pressure Urticaria; IVIG, Intravenous Immunoglobulin.

^a^ Has also H_1_ and H_2_‐antihistaminergic properties usually effective at a dose of 50–75 mg daily [26].

^b^ Does include low histamine diet as pseudoallergen‐free diet is also low in histamine. The evidence is controversial and different interpretations about what a pseudoallergen is or what foods contain them exist.

^c^ No longer available in most countries; alternative H2‐antihistamines are available including famotidine and nizatidine but evidence for their use in chronic urticaria varies.

^d^ Treatment can be considered especially if CSU and DPU are co‐existent in a patient.

^e^ Treatment can be considered especially if CSU and eosinophilic asthma are co‐existent in a patient.

H_2_‐antagonists, leukotriene antagonists, hydroxychloroquine, methotrexate, and dapsone, discussed in previous versions of this guideline as well as in national guidelines [212], are now perceived to have too little evidence to maintain them as recommendable in the algorithm, but they may still have relevance as they are very affordable in some more restricted health care systems. Sulfasalazine, interferon, plasmapheresis, phototherapy, intravenous immunoglobulins (IVIG/IGIV) and other treatment options have low‐quality evidence or just case series have been published (Table 12) [213]. Despite the lack of published evidence, all these drugs may be of value to individual patients in the appropriate clinical context [214]. This circumstance is already now reflected in available national guidelines that might justifiably deviate from the treatment algorithm outlined here, such as the current guideline for the diagnosis and management of urticaria in Indian settings [212]. This international guideline indeed encourages national adaptations but also recommends that deviations from the evidence proven treatment algorithm should be discussed with the patient as the above mentioned alternatives are not licensed for the treatment of urticaria.

Further information as well as a systematic presentation of the processed evidence underlying the treatment recommendation can be found in the evidence report (pp. 43–48).

**Table:** 

**Are H**_**2**_ **‐antihistamines useful as add‐on treatment in patients unresponsive to low or high doses of H**_**1**_ **‐antihistamines?**		
We **cannot make a recommendation** for or against the combined use of H_1_‐ and H_2_‐antihistamines in patients with chronic urticaria.	**0**	Consensus^1^ Evidence‐ and consensus‐based (see Evidence Report, pp. 43–48)
^1^> 75% agreement		

Anti‐IL‐23 [215] and antagonists of tumor necrosis factor alpha (TNF‐alpha) [216] and IVIG [217, 218, 219] which have been successfully used in case reports, are recommended currently only to be used in specialized centers as a last option (i.e., anti‐TNF‐alpha for delayed pressure urticaria and IVIG for CSU) [220, 221].

For the treatment of CSU and symptomatic dermographism, UV‐B (narrow band‐UVB, TL01), UV‐A and PUVA treatment for 1–3 months can be added to antihistamine treatment [222, 223, 224] but caution should be taken regarding the carcinogenic properties of UV light treatment.

Some treatment alternatives formerly proposed have been shown to be ineffective in double‐blind, placebo‐controlled studies and should no longer be used as the certainty of the evidence is low. These include tranexamic acid and sodium cromoglycate in CSU [225, 226], nifedipine in symptomatic dermographism/urticaria factitia [227], and colchicine and indomethacin in delayed pressure urticaria [228, 229]. However, more research may be needed for patient subgroups, for example, a pilot study [230] of patients with elevated D‐dimer levels showed heparin and tranexamic acid therapy may be effective.

**Table:** 

**Could any other treatment options be recommended for the treatment of urticaria?**		
We **cannot make a recommendation** with respect to further treatment options as standard therapies, but these may be considered in special cases with comorbidities or where financial, legal, or other limitations for the recommended algorithm treatment exist.	**0**	Consensus^1^ Expert consensus
^1^> 75% agreement		

### 5.5 Treatment of Special Populations

#### 5.5.1 Children

Many clinicians use 1st generation H_1_‐antihistamines as their first choice treatment of children with urticaria assuming that their safety profile is better known than that of the modern 2nd generation H_1_‐antihistamines due to a longer experience with them, although 1^st^ generation H_1_‐antihistamines have a proven detrimental effect on school performance [231]. Also, the use of modern 2nd generation H_1_‐antihistamines is not licensed for use in children less than 6 months of age in many countries. However, 1^st^ generation H_1_‐antihistamines have an inferior safety profile compared with 2nd generation H_1_‐antihistamines, and are, therefore, not recommended as first line treatment in children with urticaria. Second‐generation H_1_‐antihistamines with proven efficacy and safety in the pediatric population include bilastine [226], cetirizine [227], desloratadine [228, 229], fexofenadine [230], levocetirizine [232], loratadine [227], and rupatadine [233].

The choice of which 2nd generation H_1_‐antihistamines to use in children with urticaria should take into consideration age and availability as not all are available as syrup or fast dissolving tablets, suitable for children. The lowest licensed age also differs from country to country. However, some are available already at infants' age. All further steps should be based on individual considerations and be taken carefully as updosing of antihistamines and further treatment options are not well studied in children. However, omalizumab is used for treating asthma (licensed from 6 years of age or older), dupilumab for treating atopic eczema (licensed from 6 months of age or older), and ciclosporin is also used in very young children in transplant medicine. In addition, a short course of corticosteroids as advised in the algorithm should be used only as a very restricted measure in children. Currently, omalizumab is the only approved biologic for anti‐histamine refractory CSU in patients aged older than 12 years, while dupilumab is undergoing trials in children ≥ 2 years with uncontrolled CSU (LIBERTY‐CSU CUPIDKids; https://clinicaltrials.gov/study/NCT05526521) [234].

**Table:** 

**Should the same treatment algorithm be used in children?**		
We **suggest** using the same treatment algorithm with caution (e.g., according to licensing status, experience for use in children, weight‐ and age‐adjusted dosage) in children with chronic urticaria.	↑	Strong consensus^1^ Expert consensus
^1^> 95% agreement		

#### 5.5.2 Pregnant and Lactating Women

The same considerations in principle apply to pregnant and lactating women. In general, use of any systemic treatment should generally be avoided in pregnant women, especially in the first trimester. On the other hand, pregnant women have the right to the best therapy possible. While the safety of treatment has not been systematically studied in pregnant women with urticaria, it should be pointed out that the possible negative effects of increased levels of histamine receptor binding occurring in urticaria have also not been studied in pregnancy, whereas emergency referrals due to urticaria exacerbations during pregnancy have been associated with preterm birth in the PREG‐CU study. Regarding treatment, no reports of birth defects in women having used modern 2nd generation H_1_‐antihistamines during pregnancy have been reported to date. However, only small sample size studies are available for cetirizine and one large meta‐analysis for loratadine [235, 236]. Furthermore, as several modern 2nd generation H_1_‐antihistamines are now prescription free and used widely in both allergic rhinitis and urticaria, it must be assumed that many women have used these drugs especially in the beginning of pregnancy, at least before the pregnancy was confirmed. Nevertheless, since the highest safety is mandatory in pregnancy, the suggestion for the use of modern 2nd generation H_1_‐antihistamines is to prefer loratadine with the possible extrapolation to desloratadine and cetirizine with a possible extrapolation to levocetirizine. All H_1_‐antihistamines are excreted in breast milk in low concentrations. Use of 2nd generation H_1_‐antihistamines is advised, as nursing infants occasionally develop sedation from the old 1st generation H_1_‐antihistamines transmitted in breast milk.

The increased dosage of modern 2nd generation H_1_‐antihistamines can only be carefully suggested in pregnancy since safety studies have not been done, and with loratadine it must be remembered that this drug is metabolized in the liver which is not the case for its metabolite desloratadine. 1st generation H_1_‐antihistamines should be avoided [133]. The use of omalizumab in pregnancy has been reported to be safe and to date there is no indication of teratogenicity [237, 238, 239, 240]. All further steps should be based on individual considerations, with a preference for medications that have a satisfactory risk‐to‐benefit ratio in pregnant women and neonates with regard to teratogenicity and embryotoxicity. For example, ciclosporin, although not teratogenic, is embryotoxic in animal models and is associated with preterm delivery and low birth weight in human infants. Whether the benefits of ciclosporin in CU are worth the risks in pregnant women will have to be determined on a case‐by‐case basis. However, all decisions should be re‐evaluated according to the current recommendations published by regulatory authorities.

**Table:** 

**Should the same treatment algorithm be used in pregnant women and during lactation?**		
We **suggest** using the same treatment algorithm with caution both in pregnant and lactating women after risk‐benefit assessment. Drugs contraindicated or not suitable in pregnancy should not be used.	↑	Strong consensus^1^ Expert consensus
^1^> 95% agreement		

## 6 Need for Further Research

The panel and participants highlighted several key areas where additional research is needed. These include various aspects of urticaria such as its pathophysiology, epidemiology, and treatment approaches. An overview of the identified research gaps is provided in Table 13.

**TABLE 13:** Areas of further research in urticaria.

Global epidemiology, in adults and children The socioeconomic consequences, particularly in developing countries Identification of mast cell/basophil activating factors Identification of new histological markers Identification of serum biomarkers of urticarial activity/mast cell activation Clarification of the role of coagulation/coagulation factors in CSU Development of commercially available in vitro tests for detecting serum auto‐antibodies for anti‐IgE and anti‐FcɛRI Further evaluation of IgE‐ and IgG auto‐antibodies Clarification of associated psychiatric/psychosomatic diseases and their impact Pathomechanisms in antihistamine‐resistant urticaria/angioedema Double blind controlled trials comparing different modern 2nd generation H_1_‐antihistamines in higher doses in CSU and different subtypes of urticaria Safety profile of available treatments, long‐term pharmacosurveillance Multicenter studies on the possible effect of anticoagulants (oral and heparin derivatives) on CSU Controlled multicenter trials on the possible effect of add‐on of H_2_‐antihistamines, montelukast, sulfones (dapsone/sulfasalazine), methotrexate, azathioprine, and Define and achieve consensus on key concepts such as complete control, remission, relapse, or disease modification Validation of clinical and serum biomarkers that predict therapeutic response to antihistamines, omalizumab, ciclosporin, and future CU drugs Development of better treatment options for patients with difficult‐to‐treat endotypes, including disease‐modifying treatments Investigation of molecular and cellular factors associated with urticaria remission Neuro‐immuno‐psychological mechanisms Trials and licensing of 2nd generation H_1_‐antihistamines for the treatment of children below 6 months of age

## Funding

This work was supported by the British Society for Allergy & Clinical Immunology (BSACI), Gulf Academy of Allergy and Clinical Immunology (GA2CI), Asia Pacific Association of Allergy, Asthma and Clinical Immunology (APAAACI), American Academy of Dermatology, Global Allergy and Asthma Excellence Network (GA²LEN), and European Dermatology Forum (EDF).

## Disclosure

As this is an international guideline, no comment is given regarding the licensing of the drugs mentioned for the treatment of urticaria. It is in the duty of the treating physician to adhere to the relevant local regulations.

## Conflicts of Interest

An overview of the declarations of personal–financial conflicts of interest of all authors/members of the expert panel is given in the Methods Report, which is available on the EDF website: https://www.guidelines.edf.one/edf‐guidelines‐and‐consensus‐statements.

## Data Availability

Data sharing not applicable to this article as no datasets were generated or analyzed during the current study.
